# The Proton Dissociation of Bio-Protic Ionic Liquids: [AAE]X Amino Acid Ionic Liquids

**DOI:** 10.3390/molecules26010062

**Published:** 2020-12-25

**Authors:** Ting He, Cheng-Bin Hong, Peng-Chong Jiao, Heng Xiang, Yan Zhang, Hua-Qiang Cai, Shuang-Long Wang, Guo-Hong Tao

**Affiliations:** 1 Institute of Chemical Materials, China Academy of Engineering Physics, Mianyang 621900, China; heting0221@caep.cn (T.H.); jiaopengchong@163.com (P.-C.J.); xiangheng@caep.cn (H.X.); zhang_yan@caep.cn (Y.Z.); 2 College of Chemistry, Sichuan University, Chengdu 610064, China; hong_c_b@163.com (C.-B.H.); 2016141231241@stu.scu.edu.cn (S.-L.W.)

**Keywords:** protic ionic liquids, Brønsted acidity, amino acid ionic liquids, bio-based ionic liquids

## Abstract

[AAE]X composed of amino acid ester cations is a sort of typically “bio-based” protic ionic liquids (PILs). They possess potential Brønsted acidity due to the active hydrogens on their cations. The Brønsted acidity of [AAE]X PILs in green solvents (water and ethanol) at room temperature was systematically studied. Various frameworks of amino acid ester cations and four anions were investigated in this work from the viewpoint of structure–property relationship. Four different ways were used to study the acidity. Acid dissociation constants (p*K*_a_) of [AAE]X determined by the OIM (overlapping indicator method) were from 7.10 to 7.73 in water and from 8.54 to 9.05 in ethanol. The p*K*_a_ values determined by the PTM (potential titration method) were from 7.12 to 7.82 in water. Their Hammett acidity function (*H*_0_) values (0.05 mol·L^−1^) were about 4.6 in water. In addition, the p*K*_a_ values obtained by the DFT (proton-transfer reactions) were from 7.11 to 7.83 in water and from 8.54 to 9.34 in ethanol, respectively. The data revealed that the cationic structures of [AAE]X had little effect and the anions had no effect on the acidity of [AAE]X. At the same time, the OIM, PTM, Hammett method and DFT method were reliable for determining the acidic strength of [AAE]X in this study.

## 1. Introduction

Protic ionic liquids (PILs) are an important subset of ionic liquids (ILs). PILs possess strong dissolvabilities, high thermal stabilities, designable structures and broad electrochemical windows [1,2]. They play important roles in fuel cells, electrochemistry, liquid-liquid extraction, gas capture, biological media and so on due to their acidity [3,4,5,6,7]. For the existence of active hydrogen in amino acid cations, proton dissociation occurs in different solvents to varying degrees [8]. Therefore, PILs have been considered as acidic catalysts for the replacement of hazardous acids in many catalytic reactions, including the esterification reaction, biomass conversion, transformation of CO_2_ and Diels-Alder reaction. [9,10,11,12].

Bio-based ILs have been paid more attention in recent years due to their preferable green characters [13]. Some natural materials, including carboxylate salts, amino acids and sugars or sugar derivatives, have been employed as IL precursors in a green way [14]. Among these natural materials, amino acids and their derivatives are the most abundant natural sources containing quaternary nitrogens. Amino acid ionic liquids (AAILs) are fascinating for chemists in view of their close associations with chirality and biomolecules [15,16]. Some research has found that AAILs may be useful as potential solvents, catalysts, absorbents and selectors, etc. [17,18]. At the same time, AAILs can be used as acidic catalysts in the esterification of renewable valeric acid, styrene carbonate synthesis under CO_2_, the alkylation of indoles and so on [19,20,21]. Moreover, [AAE]X (AAE means the amino acid ester cations, and X means the corresponding anions) AAILs have higher thermostabilities and lower melting points, as well as lower viscosities than those of their [AA]X (cations are amino acid) analogs and, hence, broader prospects in acid-involving processes [22].

In general, water and ethanol are considered as green media for acidic catalytic reactions, which is one of the “twelve principles” of green chemistry [23,24,25,26]. Acidic properties in solvents are very important to industrially relevant reactions [27,28]. The common PILs, such as imidazolium salts and quaternary ammonium salts, have been studied in many acidic catalytic reactions in solvents [29,30]. However, the precursors of [AAE]X are more abundant and bio-based compared to common PILs, which also possesses Brønsted acidity. Therefore, the proton dissociation of [AAE]X ILs in green solvents are interesting and important for their applications. However, the acidic characteristics of [AAE]X AAILs in solvents are still lacking. The acid dissociation constant (p*K*_a_) is one of the most significant physiochemical parameters. An accurate p*K*_a_ value is important to select reaction conditions in catalytic chemistry [27,31,32,33]. Determining the acidity of ILs in water and ethanol has become an intriguing topic, since water and ethanol are promising media for green chemistry [30,34,35,36,37]. Herein, four different methods were employed to study the Brønsted acidity of [AAE]X PILs with different amino acid ester cations and anions in water and ethanol carried out.

## 2. Results

### 2.1. Overlapping Indicator Method (OIM)

**Scheme 1:**
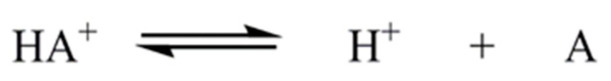
The acid dissociation reaction of protic ionic liquids (PILs).

The overlapping indicator method (OIM) is a mature method to determine the p*K*_a_ values [38,39]. The acid dissociation reaction of [AAE]X in water and ethanol can be simplified by the expression in Scheme 1.

**Scheme 2:**
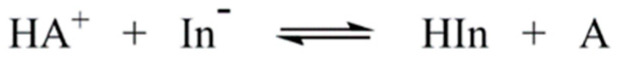
The reactions of the determinant (HA) and indicator (In^−^).

The reactions of the determinant (HA^+^) and indicator (In^−^) can be described by Scheme 2.

The chemical equilibrium constant (*K*_a_) can be obtained by the equation:

(1) $$K_{a}=\frac{K_{a}{(\mathrm{HA}}^{+})}{K_{a}\left(\mathrm{HIn}\right)}=\frac{\left[A\right]\left[\mathrm{HIn}\right]}{\left[{\mathrm{HA}}^{+}\right]\left[{\mathrm{In}}^{-}\right]},$$

The acidic dissociation constant (*K*_a_) of HA^+^ can be written as:

(2) $$pK_{a}({\mathrm{HA}}^{+})=pK_{a}\left(\mathrm{HIn}\right)-\mathrm{lg}\frac{\left[A\right]\left[\mathrm{HIn}\right]}{\left[{\mathrm{HA}}^{+}\right]\left[{\mathrm{In}}^{-}\right]},$$

where p*K*_a_ (HIn) is the p*K*_a_ value of the 4-nitrophenol indicator in water (7.15) and 2,4-dinitrophenol indicator in ethanol (8.21) [40,41]. It is easy to get the relationship between the UV/Vis absorption intensity and concentration of the indicator by the Lambert Beer law. Therefore, the concentrations of HIn, In^−^, HA^+^ and A can be measured by the absorbed change of the indicator after adding the quantitative determinant.

**Figure 1:**
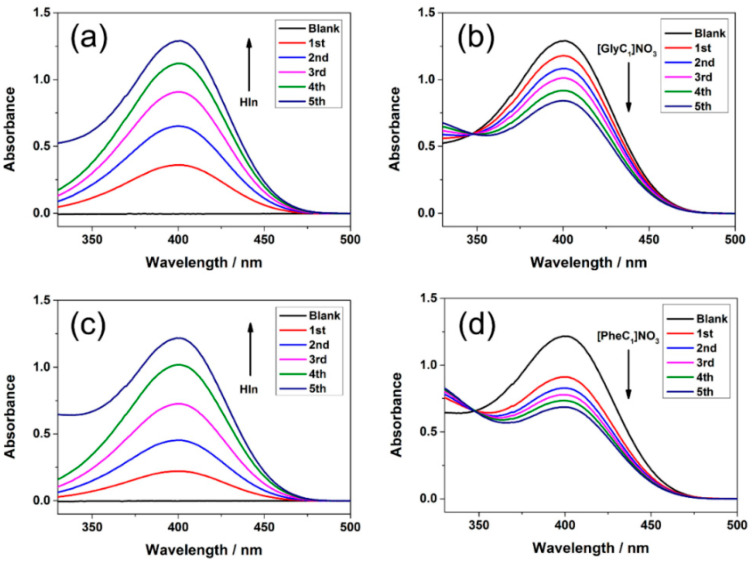
Absorption spectra of 4-nitrophenolate anion for various adding amounts of the indicator or [AAE]X during the titration in water. (**a**) Adding the indicator before the titration of [GlyC_1_]NO_3_. (**b**) Adding [GlyC_1_]NO_3_ during the titration. (**c**) Adding the indicator before the titration of [PheC_1_]NO_3_. (**d**) Adding [PheC_1_]NO_3_ during the titration.

The p*K*_a_ value is a quantitative parameter to insure the strength of the Brønsted acids. The lower p*K*_a_ values means the stronger acidity of the PILs. The UV/Vis spectral absorbance of the indicator (sodium 4-nitrophenolate) after every titration in water is illustrated in Figure 1. (The other UV-Vis spectra of the titration of [AAE]X is illustrated in Figures S1–S18). Figure 1a,c respectively represent the absorption spectra of the indicator after adding a quantitative indicator to the alkali liquor (sodium hydroxide). Figure 1b,d respectively represent the absorption spectra of the indicator after adding the quantitative [AAE]X of [GlyC_1_]NO_3_ and [PheC_1_]NO_3_. The absorption intensity at the maximum absorption wavelength of the 4-nitrophenolate anion continually decreased after quantificationally adding [AAE]X in the solvents. The p*K*_a_ values were obtained by the change of the absorption intensity at the maximum absorption wavelength. The p*K*_a_ values of [AAE]X and the contrastive compounds in water by OIM according to Equation (2) are listed in Table 1.

To obtain the influence of the anions on the Brønsted acidity, Cl^−^, NO_3_^−^, NTf_2_^−^ and ClO_4_^−^ were checked. The impact of the conformation of the cations on the Brønsted acidity of [AAE]X was also studied by the OIM. (Table 2)

### 2.2. Potential Titration Method (PTM)

To confirm the accuracy of the p*K*_a_ values determined by the OIM, the potential titration method (PTM) was also used to measure the p*K*_a_ as a comparative method [46]. All solutions are electrically neutral, i.e., the sum of all positive charges must equal the sum of all negative charges; thus,

[HA^+^] + [K^+^] + [H^+^] = [OH^−^] + [X^−^]. (3)

Since all salts are considered as being completely ionized, [K^+^] equals the concentration of potassium hydroxide (after considering the dilution by the solution). Hence,

[HA^+^] + [KOH] + [H^+^] = [OH^−^] + [X^−^]. (4)

The total concentration of acid taken is present in two forms, HA^+^ and A. Consequently,

*c*_0_ = [X^−^] = [HA^+^] + [A]. (5)

By combining Equation (4),

[HA^+^] = *c*_0_ + [OH^−^] − [KOH] − [H^+^]. (6)

In these equations, [KOH] represents the concentration that the alkali would achieve by dilution if no other substance was present in the solution. The concentration of [AAE]X is 0.100 mol·L^−1^, so we can ignore the activity coefficient. Then p*K*_a_ can be obtained by combining Equations (3) and (10):

(7) $$pK_{a}(\mathrm{HA})=\;-\mathrm{lg}(\frac{a_{\left(H^{+}\right)}a_{\left(A\right)}}{a_{{(\mathrm{HA}}^{+})}})\;=\;\mathrm{pH}-\frac{c_{0}-[{\mathrm{HA}}^{+}]}{\left[{\mathrm{HA}}^{+}\right]}.$$

The p*K*_a_ values of [AAE]X by PTM are presented in Table 3.

### 2.3. pK_a_ Values Measured by the OIM in Ethanol

**Figure 2:**
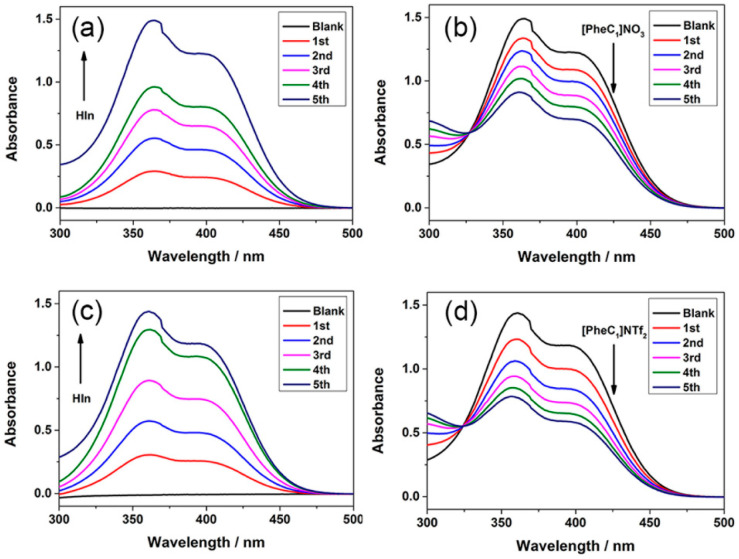
Absorption spectra of the 2,4-dinitrophenolate ion for various adding amounts of the indicator or [AAE]X during the titration in ethanol. (**a**) Adding the indicator before the titration of [PheC_1_]NO_3_. (**b**) Adding [PheC_1_]NO_3_ during the titration. (**c**) Adding the indicator before the titration of [PheC_1_]NTf_2_. (**d**) Adding [PheC_1_]NTf_2_ during the titration.

To ulteriorly study the acidity of [AAE]X, the p*K*_a_ values in ethanol were measured by the OIM with 2,4-dinitrophenol as the indicator. The principle of measuring the p*K*_a_ values in ethanol is the same to that in water. The UV/Vis spectral absorbances of sodium 2,4-dinitrophenolate after titration every time in ethanol are illustrated in Figure 2. Additionally, the p*K*_a_ values of [AAE]X PILs and the contrastive compounds in ethanol are shown in Table 4.

### 2.4. Hammett Acidity

The Brønsted acidity associated with the Hammett acidity function (*H*_0_) of [AAE]X was investigated in water to confirm the acidic strength of [AAE]X determined by the PTM and OIM [49]. Sodium 2,4-dinitrophenolate was used as an indicator for the determination of the Hammett acidity function by UV/Vis spectroscopy. For insuring the Brønsted acidity of [AAE]X, the protonated extent of the charged indicator bases (sodium 2,4-dinitrophenolate) in an aqueous solution (5 × 10^−5^ mol·L^−1^), in terms of the measurable ratio [In^−^]/[HIn], needs to be evaluated. In water, the Hammett acidity function can be expressed as the equation:

(8) $$H_{0}=pK_{a}(\mathrm{HIn})\;+\mathrm{lg}(\frac{\left[{\mathrm{In}}^{-}\right]}{\left[\mathrm{HIn}\right]}),$$

where p*K*_a_ (HIn) is the p*K*_a_ value of the 2,4-dinitrophenol indicator in water (4.12) [50], and [In^−^] and [HIn] are the molar concentrations of the unprotonated and protonated forms of the 2,4-dinitrophenolate indicator, separately.

The Hammett acidity functions (*H*_0_) of some [AAE]X and the contrastive compounds in water are listed in Table 5, Tables S1 and S2.

### 2.5. pK_a_ Values Calculated by DFT

The theoretical and experimental p*K*_a_ values of [AAE]^+^ in water and ethanol are summarized in Table 6. The p*K*_a_ values of [AAE]^+^ are from 7.11 to 7.83 in water and 8.54 to 9.34 in ethanol, separately. Additionally, the p*K*_a_ values of [AAE]X determined by the OIM are from 7.10 to 7.73 in water and from 8.54 to 9.05 in ethanol, separately. The theoretical values of [AA]X obtained are consistent with their experimental values. Therefore, the DFT method is fast and convenient to calculate the acidity of [AAE]X.

## 3. Discussion

**Figure 3:**
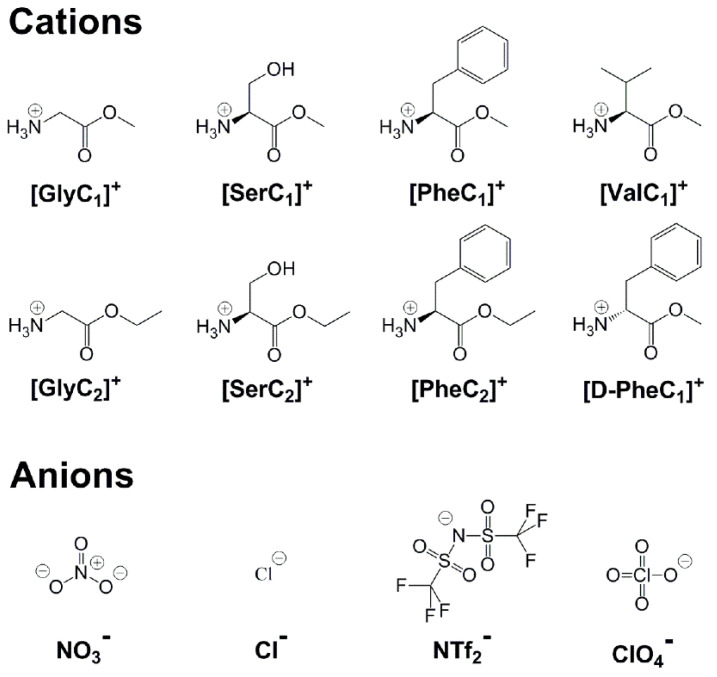
Structures and abbreviations of the [AAE]X cations and anions.

To systematically explore the acidity of [AAE]X, eight [AAE]^+^ and four anions (including nitrate (NO_3_^−^), chloride (Cl^−^), perchlorate (ClO_4_^−^) and trifluoromethanesulfonate (NTf_2_^−^)) were studied from the viewpoint of the structure–property relationship. The cations and anions of [AAE]X used in this work are shown in Figure 3. All [AAE]X PILs were synthesized and characterized by the referenced method [15]. Being convenient for studying the structure–property relationship, the glycine methyl ester cation ([GlyC_1_]^+^) was chosen as the fundamental [AAE]+ framework. Other cations, including the glycine ethyl ester cation ([GlyC_2_]^+^), serine methyl ester cation ([SerC_1_]^+^), serine ethyl ester cation ([SerC_2_]^+^), phenylalanine methyl ester cation ([PheC_1_]^+^), phenylalanine ethyl ester cation ([PheC_2_]^+^), valine methyl ester cation ([ValC_1_]^+^) and D-phenylalanine methyl ester cation ([D-PheC_1_]^+^), could be viewed as the derivatives of [GlyC_1_]^+^.

According to the p*K*_a_ values of [AAE]X and the contrastive compounds in water by the OIM, the acidic strength of [GlyC_1_]NO_3_ is the weakest in the studied [AAC_1_]X (amino acid methyl ILs) and depends on the biggest p*K*_a_ values. The acidic strengths of [ValC_1_]NO_3_, [SerC_1_]NO_3_ and [PheC_1_]NO_3_ are stronger than that of [GlyC_1_]NO_3_, maybe due to the steric effect of the side chains in [ValC_1_]NO_3_ and [PheC_1_]NO_3_ and the hydrogen bonding in [SerC_1_]NO_3_. The acidic strength of [AAE]X has little difference in the same magnitude range, since the side chain of [AAE]X may exist in hydrogen bonding or steric hindrance with the –NH_3_ group. The p*K*_a_ values of [GlyC_1_]NO_3_, [GlyC_2_]NO_3_, [SerC_1_]NO_3_, [SerC_2_]NO_3_, [PheC_1_]NO_3_ and [PheC_2_]NO_3_ are 7.67, 7.73, 7.23, 7.10, 7.20 and 7.26, respectively. The methyl ester and ethyl ester group hardly affects the acidity of [AAE]X due to the low maximum difference (0.23) between methyl ester and ethyl ester. The p*K*_a_ values of [AAE]X range from 7.10 to 7.73, which are smaller than those of glycine (Gly, 9.78), phenylalanine (Phe, 9.31), ethylammonium nitrate (EAN, 10.43), diethylammonium nitrate ([Et_2_N]NO_3_, 10.68) and triethylammonium nitrate ([Et_3_N]NO_3_, 10.55). The electron-withdrawing inductive effect of the ester group may result in the stronger acidity of [AAE]X than those of their precursors (amino acids), EAN, [Et_2_N]NO_3_ and [Et_3_N]NO_3_, whose acidity also depend on the protonated amino group.

The p*K*_a_ values of [AAE]X range from 7.10 to 7.73 in water, which are almost equal to that of imidazolium ([MIM]^+^, 7.13) salts. The acidic strength of [AAE]X is between pyridinium ([Pyri]^+^, 5.17) salts and EAN (10.43), [Et_2_N]NO_3_ (10.68) and [Et_3_N]NO_3_ (10.55).

The p*K*_a_ values of [PheC_1_]Cl, [PheC_1_]NO_3_, [PheC_1_]NTf_2_ and [PheC_1_]ClO_4_ are 7.25, 7.20, 7.24 and 7.20 by the OIM, respectively. The p*K*_a_ values of [PheC_1_]X with different anions are almost the same, to some extent. It seems like anions have insignificant effects on the acidity of [AAE]X PILs. In other words, the cation and anion of [AAE]X in water may be dissociated, because water is a typical high-polar solvent (ε = 80.100) [43]. Different from anions, the p*K*_a_ values of [L-PheC_1_]NO_3_, [D-PheC_1_]NO_3_, [L-PheC_1_]Cl and [D-PheC_1_]Cl are similar.

For the same [AAE]X, we found that the △p*K*_a_ by the PTM obtained by two different determination methods are near to zero. For example, the p*K*_a_ value of [GlyC_1_]NO_3_ determined by the OIM are the same (7.67) to that from the PTM. The data suggest that both the PTM and OIM are reliable to obtain the p*K*_a_ values of [AAE]X in water.

To ulteriorly study the acidity of [AAE]X, we measured the p*K*_a_ values in ethanol by the OIM with 2,4-dinitrophenol as the indicator. The p*K*_a_ values of [AAE]X in ethanol are between 8.54 and 9.05, which are obviously bigger than those in water (7.10 to 7.73). It may be generated by the weaker basicity of ethanol, which means that the weaker intermolecular interactions between the active hydrogens and solvent molecules lead to bigger p*K*_a_ values. The p*K*_a_ values of [GlyC_1_]NO_3_, [ValC_1_]NO_3_, [SerC_1_]NO_3_ and [PheC_1_]NO_3_ are 9.05, 8.86, 8.88 and 8.54, separately. There is also a tiny difference of the p*K*_a_ values that may be due to the side chain of [AAE]X. The p*K*_a_ values of [AAE]X are smaller than [EtNH_3_]^+^ (12.0), [Et_2_NH_2_]^+^ (10.7) and [Et_3_NH]^+^ (10.22) and bigger than [Pyri]^+^ (4.30). Therefore, the acidic strength of [AAE]X in ethanol is between [Pyri]^+^ and [EtNH_3_]^+^, [Et_2_NH_2_]^+^ and [Et_3_NH]^+^. The acidic strength of [AAE]X is slightly weaker than [MIM]^+^ (7.50) in ethanol, depending on the p*K*_a_ values.

To confirm the acidic strength of [AAE]X determined by the PTM and OIM, the Brønsted acidity associated with the Hammett acidity function (*H*_0_) of [AAE]X was investigated in water. The maximum absorption peak of sodium 2,4-dinitrophenolate decreased as the acidity of the solution increased. The *H*_0_ values of [ValC_1_]NO_3_, [PheC_1_]NO_3_ and phenylalanine (Phe) are 4.37, 4.33 and 6.44 in water, separately. This means the acidic strength of [AAE]X is almost same and is stronger than that of their precursors (amino acids). The acidity of EAN, [Et_2_N]NO_3_ and [Et_3_N]NO_3_ are so weak that the decrease of the indicator’s absorbance was not detected. The *H*_0_ of [AAE]X is almost the same as [MIM]Cl (4.35) in water. Therefore, the acidic strength of [AAE]X may be almost the same as [MIM]^+^ salt and stronger than the amino acid, EAN, [Et_2_N]NO_3_ and [Et_3_N]NO_3_. The results are matched well with the results of the p*K*_a_ values in water determined by the PTM and OIM.

**Figure 4:**
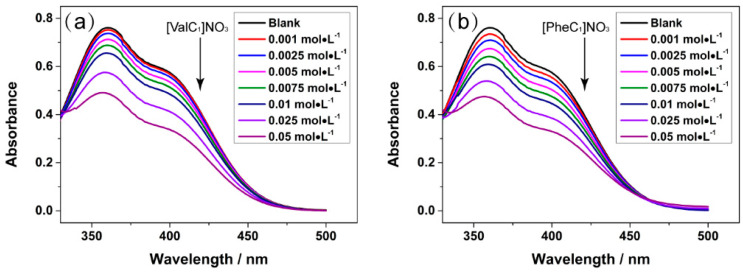
(**a**) The UV/Vis spectra of [ValC_1_]NO_3_ with different concentrations in water according to the Hammett method at 25.0 (±0.1) °C. (**b**) The UV/Vis spectra of [PheC_1_]NO_3_ with different concentrations in water according to the Hammett method at 25.0 (±0.1) °C. Indicator: sodium 2,4-dinitrophenolate 5.0 × 10^−5^ mol·L^−1^.

The relationship between the acidity and concentration is important for many applications, such as catalysis [9,37,51]. The *H*_0_ of [ValC_1_]NO_3_ and [PheC_1_]NO_3_ in aqueous solutions at various concentrations were measured. In the UV/Vis spectra, a noticeable decrease of the maximum absorption peak was found, accompanied with adding [ValC_1_]NO_3_ and [PheC_1_]NO_3_ (Figure 4). The *H*_0_ reduced when the concentration increased. The relationship between the concentration of [ValC_1_]NO_3_ and [PheC_1_]NO_3_ and the *H*_0_ can be obtained using the fitting equations. Their fitting equations are:

(9) $$H_{0}=-1.982\mathrm{lg}\left(c^{0.5}\right)+3.014\;R^{2}=0.993\;\mathrm{and}$$

(10) $$H_{0}=-1.471\mathrm{lg}\left(c^{0.5}\right)+3.319\;R^{2}=0.991.$$

**Figure 5:**
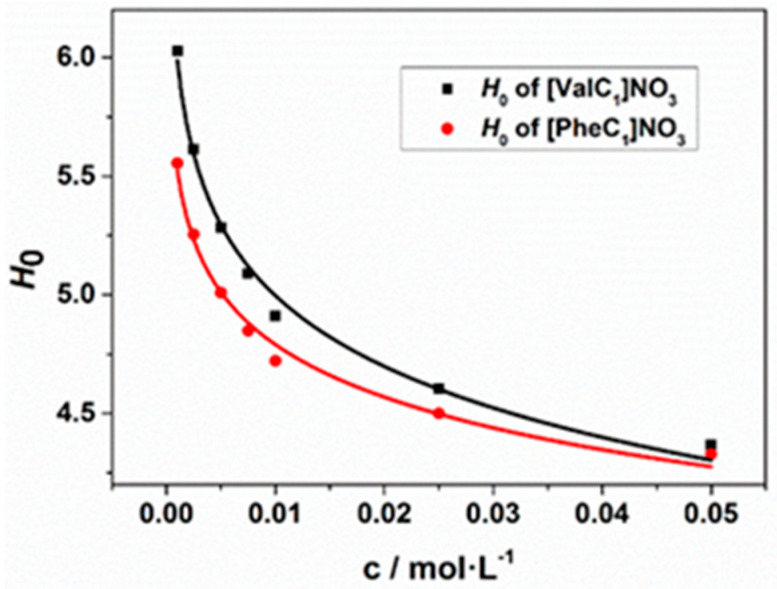
The relationship of [ValC_1_]NO_3_ and [PheC_1_]NO_3_ between the *H*_0_ and concentrations in water at 25.0 (±0.1) °C.

Their nonlinear fittings are shown in Figure 5. The *H*_0_ of [AAE]X gradually lowered with the concentration of [AAE]X rising. Based on these, the desired acidic strength can be obtained by choosing the appropriate concentrations.

## 4. Materials and Methods

General methods: All [AAE]Cl were purchased from Energy Chemical (Shanghai, China). Ethylamine (EAN) and ethanol (EtOH) were purchased from Sinopharm Chemical Reagent Co., Ltd. (Shanghai, China). All chemicals were obtained commercially as analytical-grade materials and used as received. Solvents were dried by standard procedures. [AAE]X PILs were synthesized according to a literature procedure by the ion exchange reaction of [AAE]Cl precursors with corresponding salts. The synthesized [AAE]X PILs needed to be dried firstly and kept in vacuum before use.

The standard deviation (SD) was calculated by the equation:

(11) $$\mathrm{SD}=\sqrt{\frac{{\left(A_{1}-\bar{A}\right)}^{2}+{\left(A_{2}-\bar{A}\right)}^{2}+\cdots +{\left(A_{i}-\bar{A}\right)}^{2}}{i-1}};\;\bar{A}=\frac{A_{1}+A_{2}+\cdots +A_{i}}{i}.$$

p*K*_a_ determination by the PTM: A stock solution (0.010 mol·L^−1^) of [AAE]X PILs was prepared in ultrapure water. Then, the solution was titrated with aqueous KOH solution (0.100 mol·L^−1^). The electric potential (E) (±1 mV) of the solution was obtained using an Ag–AgCl/glass combination electrode on an OHAUS Starter 2100 pH meter at 25.0 (±0.1) °C. Three standard buffer solutions with the pH values of 4.00, 6.86 and 9.18, respectively, were used to adjust the instrument before titration.

p*K*_a_ determination by OIM: The method determined the p*K*_a_ of an “unknown” acid relative to that of an “indicator” acid (whose p*K*_a_ was known) by monitoring the changes of UV/vis absorption of the indicator during titrations under standard conditions. (The indicators (In) here should show different UV/Vis absorbance between the HIn^+^ and In species. Besides, in order to produce moderate changes in the titration, the p*K*_a_ of HIn^+^ should be close to the measured substance in each solvent.) There were two steps measuring the p*K*_a_ values by the OIM. Firstly, the linear relation between the concentration of the indicator and absorbance could be achieved by adding the indicator to the alkali solution until the indicator was slightly excessive compared to the alkali. Secondly, an “unknown” acid was quantitatively added to the above solution to achieve the concentrations of HIn, In^−^, HA^+^ and A. The UV/vis absorption (A) (±0.0001) of the indicator during the titrations was obtained using a BFRL UV-1601 UV/VIS spectrophotometer 25.0 (±0.1) °C.

Hammett acidity function: The Hammett acidity function of the ILs was investigated on a BFRL UV-1601 UV/VIS spectrophotometer. Samples were measured in sealed 1-cm quartz cuvettes (Helma). The dyes of sodium 2,4-dinitrophenolate were used as the indicator and molecular probe for the determination of the *H*_0_ with 5.0 × 10^−5^ mol·L^−1^. Absorbance values of the indicator after adding acid in an aqueous solution were recorded between 330 and 500 nm at 25.0 (±0.1) °C. The concentration of [AAE]X PILs was 0.050 mol·L^−1^.

**Scheme 3:**
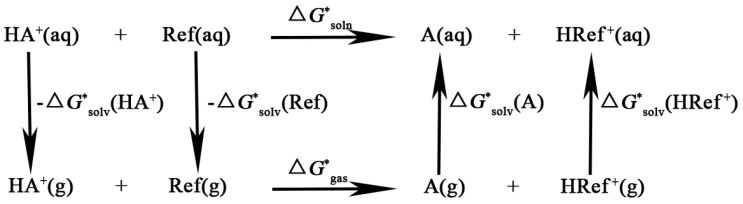
The proton-transfer reaction between [AAE]^+^ and Ref (Ref is the solvent).

Computational methods: The Brønsted acidity of [AAE]X coming from the –NH_3_ group was determined by the above experimental data. In order to obtain a better understanding of the Brønsted acidities of [AAE]X PILs, the p*K*_a_ values of [AAE]X were calculated by the density functional theory (DFT) using the Gaussian 09 suite program [52]. It was verified that the anions would have hardly any effect on the acidity of [AAE]X in water or ethanol by the experimental data. Therefore, the calculated acidity of [AAE]^+^ could be considered as a simple and approximate method for determining the acidity of [AAE]X. The p*K*_a_ values by theoretical calculation could be carried out by using the proton-transfer reaction (Scheme 3) [53,54]:

where the solution-free energy was calculated by:

(12) $$△G_{\mathrm{sol}}^{*}=△G_{g}^{*}+△G_{\mathrm{sol}}^{*}\left({\mathrm{HRef}}^{+}\right)+△G_{\mathrm{sol}}^{*}\left(\mathrm{AAE}\right)-△G_{\mathrm{sol}}^{*}\left({\left[\mathrm{AAE}\right]}^{+}\right)-△G_{\mathrm{sol}}^{*}\left(\mathrm{Ref}\right).$$

Then, it led to the following equilibrium in Equation (13):

(13) $$K_{a}=\frac{{[\mathrm{AAE}][\mathrm{HRef}}^{+}]}{\left[{\left[\mathrm{AAE}\right]}^{+}\right]}=e^{\frac{-△G_{\mathrm{sol}}^{*}}{\mathrm{RT}}}.$$

The calculation of the p*K*_a_ was obtained from Equation (14):

(14) $$pK_{a}\left({\left[\mathrm{AAE}\right]}^{+}\right)=\frac{△G_{\mathrm{sol}}^{*}}{2.303\mathrm{RT}}-\mathrm{lg}\left(\mathrm{Ref}\right).$$

The final expression for the p*K*_a_ can be written as

(15) $$pK_{a}\left({\left[\mathrm{AAE}\right]}^{+}\right)=\frac{△G_{\mathrm{sol}}^{*}}{2.303\mathrm{RT}}-K,$$

where K was a correction value dependent on the experimental values. The structures of [AAE]^+^, AAE, H_2_O, H_3_O^+^, Et_2_OH and Et_2_OH_2_^+^ were optimized in the gas phase at the B3LYP/6-311++G(d,p) level [55,56,57]. On the basis of the optimized structures, the solvation-free energies of [AAE]^+^ and AAE in water or ethanol were calculated with the polarizable continuum model (PCM) at the B3LYP/6-311++G(d,p) level [53,58]. The liquidus Gibbs-free energies of AAEH^+^ and AAE were obtained from the sum of the total electronic energies in water or ethanol and the thermal corrections to the gaseous Gibbs-free energies (G_corr_).

## 5. Conclusions

The proton dissociation of [AAE]X PILs as a kind of Bio-PIL was systematically studied in green solvents, water and ethanol for the first time. The p*K*_a_ values of [AAE]X PILs were from 6.99 to 7.52 in water and from 8.54 to 9.05 in ethanol by the OIM, respectively. The acidity of [AAE]X determined by the PTM, Hammett method and DFT method coincided with those by the OIM. All the methods revealed that the acidic strength of [AAE]X was the almost same to [MIM]^+^ and between [Pyri]^+^ and [EtNH_3_]^+^ in water. Additionally, the acidic strength of [AAE]X was slightly weaker than [MIM]^+^ and between [Pyri]^+^ and [EtNH_3_]^+^ in ethanol. The certain Brønsted acidity of the bio-PILs [AAE]X will help them to be considered as feasible acidic catalysts with green and recoverable features. This insight into the proton dissociation will prompt PILs being applied widely.

## Figures and Tables

**Table 1:** The acid dissociation constant (p*K*_a_) values of [AAE]X and the contrastive compounds in water.

Compound	p*K*_a_	SD
[GlyC_1_]NO_3_	7.67	0.02
[GlyC_2_]NO_3_	7.73	0.02
[ValC_1_]NO_3_	7.54	0.03
[SerC_1_]NO_3_	7.23	0.04
[SerC_2_]NO_3_	7.10	0.02
[PheC_1_]NO_3_	7.20	0.01
[PheC_2_]NO_3_	7.26	0.04
[MIM]+	7.13 (a)	/
Gly	9.78 (b)	/
Phe	9.31 (b)	/
EAN	10.43 (c)	/
[Et_2_N]NO_3_	10.68 (c)	/
[Et_3_N]NO_3_	10.55 (c)	/
[Pyri]^+^	5.17 (d)	/

Conditions: 25.0 (±0.1 °C). (a) Reference [42]. (b) Reference [43]. (c) Reference [44]. (d) Reference [45]. SD: standard deviation.

**Table 2:** The p*K*_a_ values of [AAE]X with different anions and cations.

Compound	p*K*_a_					Average p*K*_a_	SD
	1	2	3	4	5		
[PheC_1_]Cl	7.25	7.26	7.26	7.25	7.24	7.25	0.01
[PheC_1_]NO_3_	7.21	7.20	7.21	7.21	7.20	7.20	0.01
[PheC_1_]NTf_2_	7.23	7.25	7.24	7.24	7.23	7.24	0.01
[PheC_1_]ClO_4_	7.21	7.20	7.16	7.20	7.21	7.20	0.02
[PheC_1_]Cl	7.25	7.26	7.26	7.25	7.24	7.25	0.01
[D-PheC_1_]Cl	7.17	7.16	7.18	7.16	7.18	7.17	0.01
[PheC_1_]NO_3_	7.21	7.20	7.21	7.21	7.20	7.20	0.01
[D-PheC_1_]NO_3_	7.16	7.15	7.18	7.20	7.18	7.17	0.02

**Table 3:** The p*K*_a_ values of a part of [AAE]X by the overlapping indicator method (OIM) and potential titration method (PTM).

Compound	p*K*_a_		SD
	OIM	PTM	
[GlyC_1_]NO_3_	7.67	7.67	0.00
[GlyC_2_]NO_3_	7.73	7.82	0.09
[PheC_1_]NO_3_	7.20	7.19	0.01
[PheC_2_]NO_3_	7.26	7.21	0.05
[PheC_1_]Cl	7.25	7.19	0.06
[SerC_1_]NO_3_	7.23	7.24	0.01
[PheC_1_]NTf_2_	7.24	7.12	0.12

Conditions of PTM: [AAE]X (0.100 mol·L^−1^) and KOH (0.500 mol·L^−1^), 25.0 (±0.1) °C.

**Table 4:** The p*K*_a_ values of [AAE]X and the contrastive compounds in ethanol.

Compound	p*K*_a_	SD
[GlyC_1_]NO_3_	9.05	0.02
[ValC_1_]NO_3_	8.86	0.04
[SerC_1_]NO_3_	8.88	0.03
[PheC_1_]NO_3_	8.54	0.01
[PheC_2_]NO_3_	8.61	0.03
[PheC_1_]NTf_2_	8.61	0.03
[D-PheC_1_]NO_3_	8.59	0.01
[EtNH_3_]^+^	12.0 (a)	/
[Et_2_NH_2_]^+^	10.7 (a)	/
[Et_3_NH]^+^	10.22 (b)	/
[MIM]^+^	7.50 (b)	/
[Pyri]^+^	4.30 (a)	/

Conditions: 25.0 (±0.1 °C). (a) Reference [47]. (b) Reference [48].

**Table 5:** The Hammett acidity functions (*H*_0_) and p*K*_a_ values of some [AAE]X and the contrastive compounds in water.

Compound	*H*_0_	p*K*_a_ (a)
[ValC_1_]NO_3_	4.37	7.54
[PheC_1_]NO_3_	4.33	7.20
Phe	6.44	9.31
[MIM]Cl	4.35	7.13 (c)
EAN	u.d. (b)	10.43
[Et_2_N]NO_3_	u.d. (b)	10.68
[Et_3_N]NO_3_	u.d. (b)	10.55

(a) Measured by the OIM. (b) Undetected. (c) Reference [42].

**Table 6:** The theoretical and experimental p*K*_a_ values of [AAE]^+^ in water and ethanol.

[AAE]^+^	p*K*_a_(Cal) (a)	p*K*_a_(Exp) (b)	p*K*_a_(Cal) (c)	p*K*_a_(Exp) (d)
[GlyC_1_]^+^	7.11	7.67	8.54	9.08
[GlyC_2_]^+^	7.15	7.73	8.60	/
[ValC_1_]^+^	7.83	7.54	9.34	8.85
[SerC_1_]^+^	7.40	7.23	8.96	8.88
[SerC_2_]^+^	7.69	7.10	9.27	/
[PheC_1_]^+^	7.24	7.20	8.78	8.50
[PheC_2_]^+^	7.46	7.26	9.05	8.61
[D-PheC_1_]^+^	7.24	7.17	8.78	8.58

**(a)** The calculated p*K*_a_ values in water. **(b)** The experimental p*K*_a_ values in water. **(c)** The calculated p*K*_a_ values in ethanol. **(d)** The experimental p*K*_a_ values in ethanol of [AAE]NO_3_.

## Supplementary Materials

The following are available online. Table S1. The Hammett functions for [ValC_1_]NO_3_ in water. Table S2. The Hammett functions for [PheC_1_]NO_3_ in water. Figure S1. (a) The increasing absorbance during the deprotonation of the acid indicator (4-nitrophenol) by the base. (b) The decreasing absorbance of the acid indicator anion (4-nitrophenolate) during the titration of [GlyC_1_]NO_3_ in water. Figure S2. (a) The increasing absorbance during the deprotonation of the acid indicator (4-nitrophenol) by the base. (b) The decreasing absorbance of the acid indicator anion (4-nitrophenolate) during the titration of [GlyC_2_]NO_3_ in water. Figure S3. (a) The increasing absorbance during the deprotonation of the acid indicator (4-nitrophenol) by the base. (b) The decreasing absorbance of the acid indicator anion (4-nitrophenolate) during the titration of [ValC_1_]NO_3_ in water. Figure S4. (a) The increasing absorbance during the deprotonation of the acid indicator (4-nitrophenol) by the base. (b) The decreasing absorbance of the acid indicator anion (4-nitrophenolate) during the titration of [SerC_1_]NO_3_ in water. Figure S5. (a) The increasing absorbance during the deprotonation of the acid indicator (4-nitrophenol) by the base. (b) The decreasing absorbance of the acid indicator anion (4-nitrophenolate) during the titration of [SerC_2_]NO_3_ in water. Figure S6. (a) The increasing absorbance during the deprotonation of the acid indicator (4-nitrophenol) by the base. (b) The decreasing absorbance of the acid indicator anion (4-nitrophenolate) during the titration of [PheC_1_]NO_3_ in water. Figure S7. (a) The increasing absorbance during the deprotonation of the acid indicator (4-nitrophenol) by the base. (b) The decreasing absorbance of the acid indicator anion (4-nitrophenolate) during the titration of [PheC_2_]NO_3_ in water. Figure S8. (a) The increasing absorbance during the deprotonation of the acid indicator (4-nitrophenol) by the base. (b) The decreasing absorbance of the acid indicator anion (4-nitrophenolate) during the titration of [PheC_1_]Cl in water. Figure S9. (a) The increasing absorbance during the deprotonation of the acid indicator (4-nitrophenol) by the base. (b) The decreasing absorbance of the acid indicator anion (4-nitrophenolate) during the titration of [D-PheC_1_]Cl in water. Figure S10. (a) The increasing absorbance during the deprotonation of the acid indicator (4-nitrophenol) by the base. (b) The decreasing absorbance of the acid indicator anion (4-nitrophenolate) during the titration of [D-PheC_1_]NO_3_ in water. Figure S11. (a) The increasing absorbance during the deprotonation of the acid indicator (4-nitrophenol) by the base. (b) The decreasing absorbance of the acid indicator anion (4-nitrophenolate) during the titration of [PheC_1_]NTf_2_ in water. Figure S12. (a) The increasing absorbance during the deprotonation of the acid indicator (4-nitrophenol) by the base. (b) The decreasing absorbance of the acid indicator anion (4-nitrophenolate) during the titration of [PheC_1_]ClO_4_ in water. Figure S13. (a) The increasing absorbance during the deprotonation of the acid indicator (2,4-dinitrophenol) by the base. (b) The decreasing absorbance of the acid indicator anion (2,4-dinitrophenolate) during the titration of [GlyC_1_]NO_3_ in ethanol. Figure S14. (a) The increasing absorbance during the deprotonation of the acid indicator (2,4-dinitrophenol) by the base. (b) The decreasing absorbance of the acid indicator anion (2,4-dinitrophenolate) during the titration of [ValC_1_]NO3 in ethanol. Figure S15. (a) The increasing absorbance during the deprotonation of the acid indicator (2,4-dinitrophenol) by the base. (b) The decreasing absorbance of the acid indicator anion (2,4-dinitrophenolate) during the titration of [SerC_1_]NO_3_ in ethanol. Figure S16. (a) The increasing absorbance during the deprotonation of the acid indicator (2,4-dinitrophenol) by the base. (b) The decreasing absorbance of the acid indicator anion (2,4-dinitrophenolate) during the titration of [PheC_1_]NO_3_ in ethanol. Figure S17. (a) The increasing absorbance during the deprotonation of the acid indicator (2,4-dinitrophenol) by the base. (b) The decreasing absorbance of the acid indicator anion (2,4-dinitrophenolate) during the titration of [PheC_2_]NO_3_ in ethanol. Figure S18. (a) The increasing absorbance during the deprotonation of the acid indicator (2,4-dinitrophenol) by the base. (b) The decreasing absorbance of the acid indicator anion (2,4-dinitrophenolate) during the titration of [D-PheC_1_]NO_3_ in ethanol. Figure S18. (a) The increasing absorbance during the deprotonation of the acid indicator (2,4-dinitrophenol) by the base. (b) The decreasing absorbance of the acid indicator anion (2,4-dinitrophenolate) during the titration of [PheC_1_]NTf_2_ in ethanol. Optimized geometry coordinates.

## References

[1] Plechkova N.V., Seddon K.R. (2008). Applications of ionic liquids in the chemical industry. Chem. Soc. Rev..

[2] Greaves T.L., Drummond C.J. (2015). Protic ionic liquids: Evolving structure–property relationships and expanding applications. Chem. Rev..

[3] Fernicola A., Panero S., Scrosati B., Tamada M., Ohno H. (2007). New types of brönsted acid–base ionic liquids-based membranes for applications in PEMFCs. Chem. Phys. Chem..

[4] Huang M., Weingärtner H. (2008). Protic ionic liquids with unusually high dielectric permittivities. Chem. Phys. Chem..

[5] Billard I., Ouadi A., Gaillard C. (2011). Liquid–liquid extraction of actinides, lanthanides, and fission products by use of ionic liquids: From discovery to understanding. Anal. Bioanal. Chem..

[6] Pollet P., Davey E.A., Urena-Benavides E.E., Eckert C.A., Liotta C.L. (2014). Solvents for sustainable chemical processes. Green Chem..

[7] Sjöblom M., Antonopoulou I., Jiménez I.G., Maciel A.O., Khokarale S.G., Mikkola J.P., Rova U., ChristakopouloS P. (2020). Enzyme-Assisted CO_2_ Absorption in aqueous amino acid ionic liquid amine blends. ACS Sustain. Chem. Eng..

[8] Amarasekara A.S. (2016). Acidic ionic liquids. Chem. Rev..

[9] He L., Qin S., Chang T., Sun Y., Gao X. (2013). Biodiesel synthesis from the esterification of free fatty acids and alcohol catalyzed by long-chain Brønsted acid ionic liquid. Catal. Sci. Technol..

[10] Kotadia D.A., Soni S.S. (2013). Symmetrical and unsymmetrical Brønsted acidic ionic liquids for the effective conversion of fructose to 5-hydroxymethyl furfural. Catal. Sci. Technol..

[11] Hu J., Ma J., Zhu Q., Zhang Z., Wu C., Han B. (2015). Transformation of Atmospheric CO_2_ Catalyzed by Protic Ionic Liquids: Efficient Synthesis of 2-Oxazolidinones. Angew. Chem. Int. Ed..

[12] Manna A., Kumar A. (2014). Invoking pairwise interactions in water-promoted diels–alder reactions by using ionic liquids as cosolvents. Chem. Phys. Chem..

[13] Hulsbosch J., De Vos D.E., Binnemans K., Ameloot R. (2016). Biobased ionic liquids: Solvents for a green processing industry?. ACS Sustain. Chem. Eng..

[14] Dong L., He L., Tao G., Huang M., Hu C. (2013). Theoretical enthalpies of formation of [AA]X and [AAE]X type amino acid ionic liquids. J. Chem. Eng. Data.

[15] Tao G., He L., Sun N., Kou Y. (2005). New generation ionic liquids: Cations derived from amino acids. Chem. Commun..

[16] Ohno H., Fukumoto K. (2007). Amino Acid Ionic Liquids. Acc. Chem. Res..

[17] Liu Q., Wu K., Tang F., Yao L., Yang F., Nie Z., Yao S. (2009). Amino acid ionic liquids as chiral ligands in ligand-exchange chiral separations. Chem. Eur. J..

[18] Gurkan B.E., de la Fuente J.C., Mindrup E.M., Ficke L.E., Goodrich B.F., Price E.A., Schneider W.F., Brennecke J.F. (2010). Equimolar CO_2_ absorption by anion-functionalized ionic liquids. J. Am. Chem. Soc..

[19] Dong L., He L., Tao G., Hu C. (2013). High yield of ethyl valerate from the esterification of renewable valeric acid catalyzed by amino acid ionic liquid. RSC Adv..

[20] Roshan K.R., Jose T., Kim D., Cherian K.A., Park D.W. (2014). Microwave-assisted one pot-synthesis of amino acid ionic liquids in water: Simple catalysts for styrene carbonate synthesis under atmospheric pressure of CO_2_. Catal. Sci. Technol..

[21] Shiri M. (2013). Prolinium Triflate: A protic ionic liquid which acts as water-tolerant catalyst in the alkylation of indoles. J. Iran. Chem. Soc..

[22] He L., Tao G., Parrish D.A., Shreeve J.M. (2009). Slightly viscous amino acid ionic liquids: Synthesis, properties, and calculations. J. Phys. Chem. B.

[23] Capello C., Fischer U., Hungerbuhler K. (2007). What is a green solvent? A comprehensive framework for the environmental assessment of solvents. Green Chem..

[24] Gui J., Cong X., Liu D., Zhang X., Hu Z., Sun Z. (2004). Novel Brønsted acidic ionic liquid as efficient and reusable catalyst system for esterification. Catal. Commun..

[25] Manabe K., Iimura S., Sun X., Kobayashi S. (2002). Dehydration reactions in water. Brønsted acid−surfactant-combined catalyst for ester, ether, thioether, and dithioacetal formation in water. J. Am. Chem. Soc..

[26] Qi X., Watanabe M., Aida T.M., Smith R.L. (2012). Synergistic conversion of glucose into 5-hydroxymethylfurfural in ionic liquid–water mixtures. Bioresour. Technol..

[27] Zhou H., Yang J., Ye L., Lin L., Yuan Y. (2010). Effects of acidity and immiscibility of lactam-based Brønsted-acidic ionic liquids on their catalytic performance for esterification. Green Chem..

[28] Akiyama T., Itoh J., Yokota K., Fuchibe K. (2004). Enantioselective mannich-type reaction catalyzed by a chiral brønsted acid. Angew. Chem. Int. Ed..

[29] Chiappe C., Rajamani S., D’Andrea F. (2013). A dramatic effect of the ionic liquid structure in esterification reactions in protic ionic media. Green Chem..

[30] Serrano-Ruiz J.C., Campelo J.M., Francavilla M., Romero A.A., Luque R., Menendez-Vazquez C., Garcia A.B., Garcia-Suarez E.J. (2012). Efficient microwave-assisted production of furfural from C_5_ sugars in aqueous media catalysed by Brönsted acidic ionic liquids. Catal. Sci. Technol..

[31] Barhdadi R., Troupel M., Comminges C., Laurent M., Doherty A. (2012). Electrochemical Determination of pK_a_ of N-Bases in Ionic Liquid Media. J. Phys. Chem. B.

[32] Crespo G.A., Afshar M.G., Bakker E. (2012). Direct Detection of Acidity, Alkalinity, and pH with Membrane Electrodes. Anal. Chem..

[33] Millan D., Rojas M., Santos J.G., Morales J., Isaacs M., Diaz C., Pavez P. (2014). Toward a pKa Scale of N-base Amines in Ionic Liquids. J. Phys. Chem. B.

[34] Deive F.J., Rodriguez A., Pereiro A.B., Araujo J.M.M., Longo M.A., Coelho M.A.Z., Lopes J.N.C., Esperanca J., Rebelo L.P.N., Marrucho I.M. (2011). Ionic liquid-based aqueous biphasic system for lipase extraction. Green Chem..

[35] Saravanamurugan S., Paniagua M., Melero J.A., Riisager A. (2013). Efficient Isomerization of Glucose to Fructose over Zeolites in Consecutive Reactions in Alcohol and Aqueous Media. J. Am. Chem. Soc..

[36] Mihichuk L.M., Driver G.W., Johnson K.E. (2011). Brønsted Acidity and the Medium: Fundamentals with a Focus on Ionic Liquids. Chem. Phys. Chem..

[37] Zhang L., Zhang Z., Yuan W., Zhao N., Zhu Q., He L., Tao G. (2020). Hydrogen-Bonding-Driven Ion-Pair Formation in Protic Ionic Liquid Aqueous Solution. Chem. Phys. Chem..

[38] Matthews W.S., Bares J.E., Bartmess J.E., Bordwell F.G., Cornforth F.J., Drucker G.E., Margolin Z., McCallum R.J., McCollum G.J., Vanier N.R. (1975). Equilibrium acidities of carbon acids. VI. Establishment of an absolute scale of acidities in dimethyl sulfoxide solution. J. Am. Chem. Soc..

[39] Chu Y., Deng H., Cheng J. (2007). An Acidity Scale of 1,3-Dialkylimidazolium Salts in Dimethyl Sulfoxide Solution. J. Org. Chem..

[40] Fickling M., Fischer A., Mann B.R., Packer J., Vaughan J. (1959). Hammett Substituent Constants for Electron-withdrawing Substituents: Dissociation of Phenols, Anilinium Ions and Dimethylanilinium Ions. J. Am. Chem. Soc..

[41] Mchedlov-Petrosyan N.O. (2003). Ionization and Tautomerism of Hydroxyxanthenes and Some Other Dyes in Ethanol. Russ. J. Gen. Chem..

[42] Danovich D.K., Turchaninov V.K. (1989). Basicity of azoles. 1. Investigation of total energy of pyrazole and imidazole derivatives by the partitioning method. Bull. Pol. Acad. Sci. Tech..

[43] Lide D.R. (2012). CRC Handbook of Chemistry and Physics.

[44] Zhang L., He L., Hong C., Qin S., Tao G. (2015). Brønsted acidity of bio-protic ionic liquids: The acidic scale of [AA]X amino acid ionic liquids. Green Chem..

[45] Espinosa S., Bosch E., Rosés M. (2002). Retention of ionizable compounds in high-performance liquid chromatography: 14. Acid–base pK values in acetonitrile–water mobile phases. J. Chromatogr. A.

[46] Albert A., Serjeant E.P. (1984). The Determination of Ionization Constants.

[47] Zevatskii Y.E., Samoilov D.V. (2008). Empirical method for description of solvent effect on the ionization constants of NH acids. Russ. J. Org. Chem..

[48] Gibson G.T.T., Mohamed M.F., Neverov A.A., Brown R.S. (2006). Potentiometric Titration of Metal Ions in Ethanol. Inorg. Chem..

[49] Thomazeau C., Olivier-Bourbigou H., Magna L., Luts S., Gilbert B. (2003). Determination of an Acidic Scale in Room Temperature Ionic Liquids. J. Am. Chem. Soc..

[50] Glasoe P.K. (1965). Dissociation Constants for Some Nitrophenols and Salicylic Acid in Deuterium Oxide. J. Phys. Chem..

[51] Choudhary V., Mushrif S.H., Ho H., Anderko A., Nikolakis V., Marinkovic N.S., Frenkel A.I., Sandler S.I., Vlachos D.G. (2013). Insights into the Interplay of Lewis and Brønsted Acid Catalysts in Glucose and Fructose Conversion to 5-(Hydroxymethyl)furfural and Levulinic Acid in Aqueous Media. J. Am. Chem. Soc..

[52] Frisch M.J., Trucks G.W., Schlegel H.B., Scuseria G.E., Robb M.A., Cheeseman J.R., Scalmani G., Barone V., Mennucci B., Petersson G.A. (2009). Gaussian 09.

[53] Ho J., Coote M.L. (2010). A universal approach for continuum solvent pK a calculations: Are we there yet?. Theor. Chem. Acc..

[54] Pliego J.R., Riveros J.M. (2002). Theoretical Calculation of pKa Using the Cluster−Continuum Model. J. Phys. Chem. A.

[55] Zhao Y., Schultz N.E., Truhlar D.G. (2006). Design of Density Functionals by Combining the Method of Constraint Satisfaction with Parametrization for Thermochemistry, Thermochemical Kinetics, and Noncovalent Interactions. J. Chem. Theory Comput..

[56] Lee C., Yang W., Parr R.G. (1988). Development of the Colle-Salvetti correlation-energy formula into a functional of the electron density. Phys. Rev. B.

[57] Stephens P.J., Devlin F.J., Chabalowski C.F., Frisch M.J. (1994). Ab Initio Calculation of Vibrational Absorption and Circular Dichroism Spectra Using Density Functional Force Fields. J. Phys. Chem..

[58] Marenich A.V., Cramer C.J., Truhlar D.G. (2009). Universal Solvation Model Based on Solute Electron Density and on a Continuum Model of the Solvent Defined by the Bulk Dielectric Constant and Atomic Surface Tensions. J. Phys. Chem. B.

