# Supplementary material for: The Proton Dissociation of Bio-Protic Ionic Liquids: [AAE]X Amino Acid Ionic Liquids
Source: Molecules. 2020 Dec 25;26(1):62. doi: 10.3390/molecules26010062 (PMC7795477; doi:10.3390/molecules26010062)
Supplement: Supplementary file 1 [file molecules-26-00062-s001.pdf]

Supplementary Materials

The Proton Dissociation of Bio-Protic Ionic Liquids: [AAE]X Amino Acid Ionic Liquids

Ting He [1], Cheng-Bin Hong [2], Peng-Chong Jiao [1], Heng Xiang [1], Yan Zhang [1], Hua-Qiang Cai [1,*], Shuang-Long Wang [2] and Guo-Hong Tao [2,*]

[1] Institute of Chemical Materials, China Academy of Engineering Physics, Mianyang, Sichuan 621900 China; heting0221@caep.cn (T.H.); jiaopengchong@163.com (P.-C.J.); xiangheng@caep.cn (H.X.); zhang_yan@caep.cn (Y.Z.)

[2] College of Chemistry, Sichuan University, Chengdu 610064, China; hong_c_b@163.com (C.-B.H.); 2016141231241@stu.scu.edu.cn (S.-L.W.)

* Correspondence: caihuaqiang@caep.cn (H.-Q.C.); taogh@scu.edu.cn (G.-H.T.); Tel.: 86-28-85470368

The Hammett functions for [AA]X PILs in water.

Table 1. The Hammett functions for [ValC$_1$]NO$_3$ in water.

Concentration/mol·L^{-1}	A_{max} (solvent)	A_{max} (compound)	H_0
0.0010	0.7612	0.7519	6.03
0.0025	0.7612	0.7375	5.61
0.0050	0.7612	0.7124	5.28
0.0075	0.7612	0.6875	5.09
0.0100	0.7612	0.6550	4.91
0.0250	0.7612	0.5731	4.60
0.0500	0.7612	0.4862	4.37

Table 2. The Hammett functions for [PheC$_1$]NO$_3$ in water.

Concentration/mol·L^{-1}	A_{max} (solvent)	A_{max} (compound)	H_0
0.0010	0.7612	0.7342	5.55
0.0025	0.7612	0.7092	5.25
0.0050	0.7612	0.6740	5.01
0.0075	0.7612	0.6413	4.85
0.0100	0.7612	0.6088	4.72
0.0250	0.7612	0.5371	4.50
0.0500	0.7612	0.4700	4.33

UV-Vis spectra of the titration of [AAE]X.

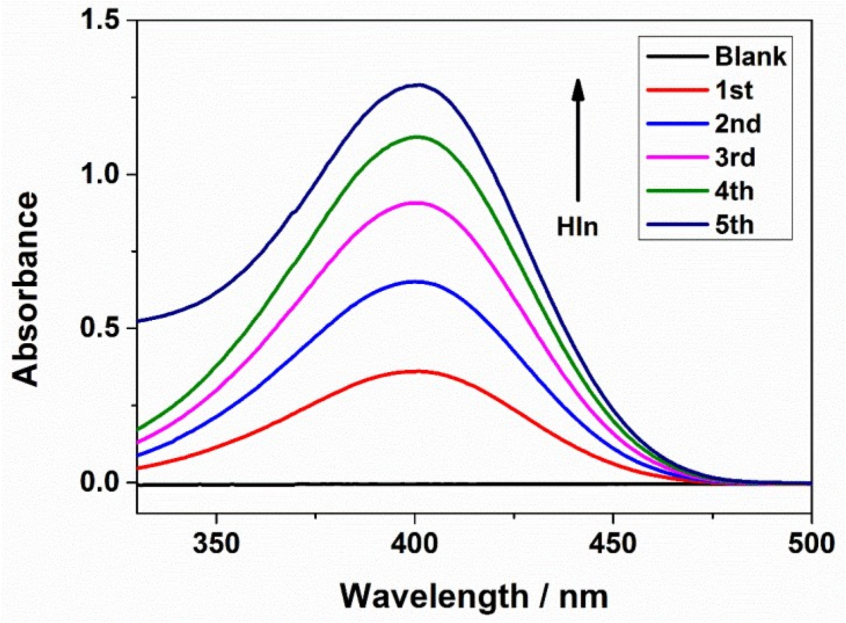

(a)

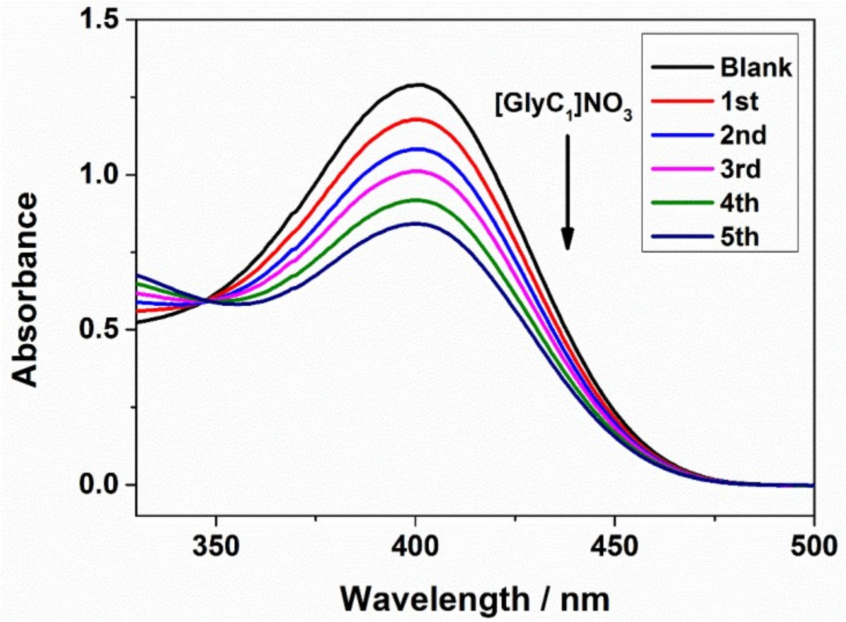

(b)

Figure S1. (**a**) The increasing absorbance during the deprotonation of the acid indicator (4-nitrophenol) by the base. (**b**) The decreasing absorbance of the acid indicator anion (4-nitrophenolate) during the titration of $[GlyC_1]NO_3$ in water.

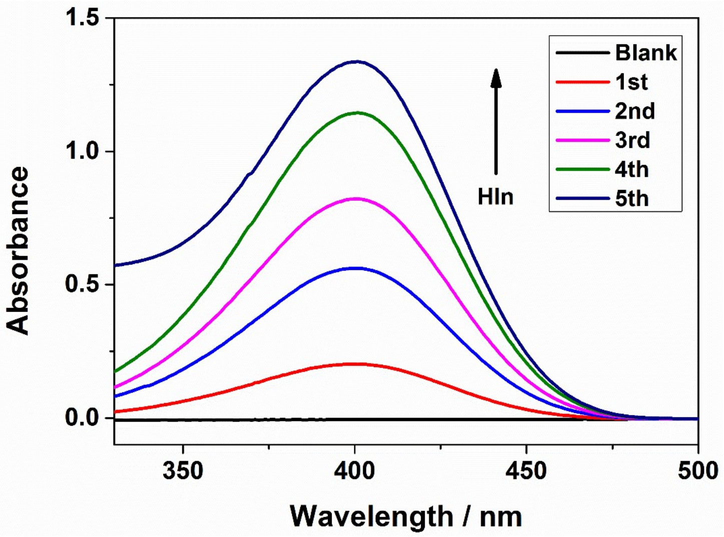

(a)

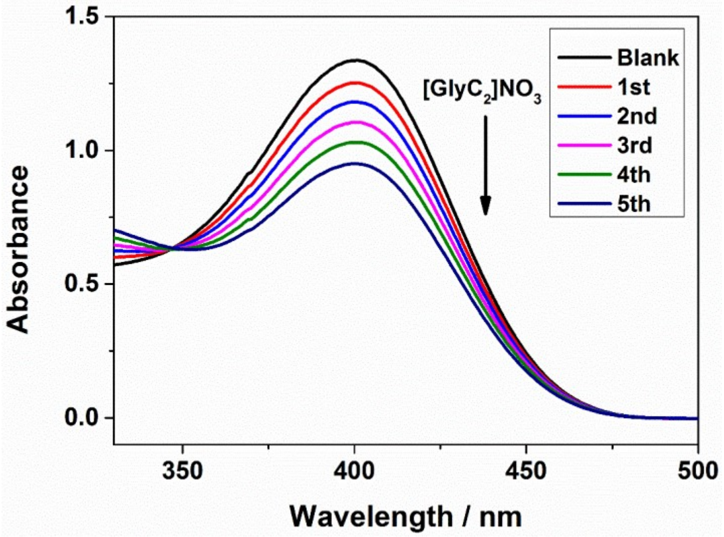

(b)

Figure S2. (**a**) The increasing absorbance during the deprotonation of the acid indicator (4-nitrophenol) by the base. (**b**) The decreasing absorbance of the acid indicator anion (4-nitrophenolate) during the titration of $[GlyC_2]NO_3$ in water.

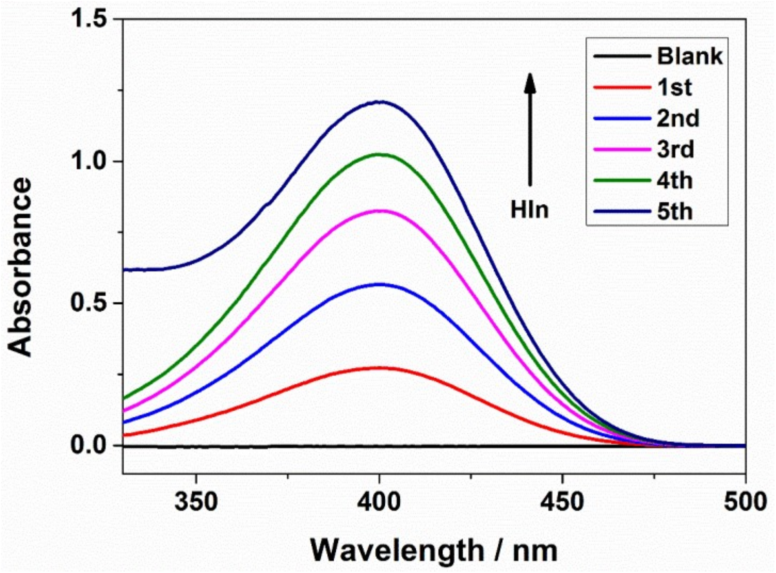

(a)

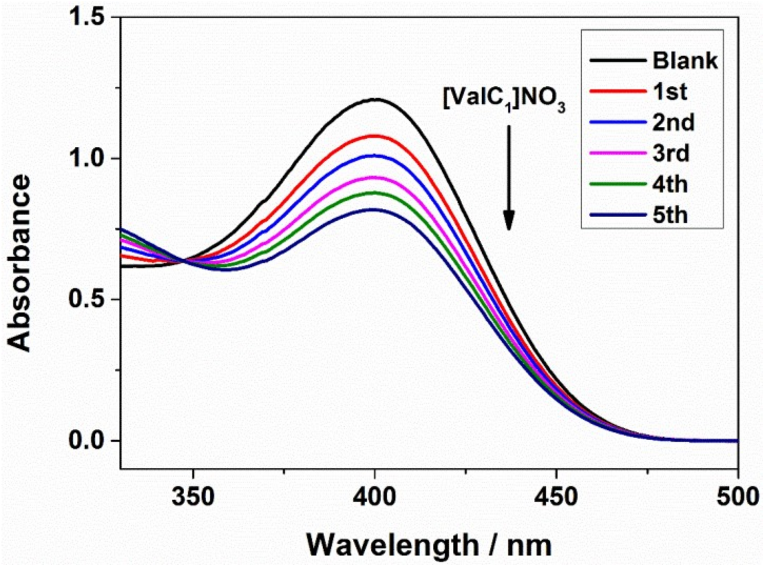

(b)

Figure S3. (**a**) The increasing absorbance during the deprotonation of the acid indicator (4-nitrophenol) by the base. (**b**) The decreasing absorbance of the acid indicator anion (4-nitrophenolate) during the titration of $[ValC_1]NO_3$ in water.

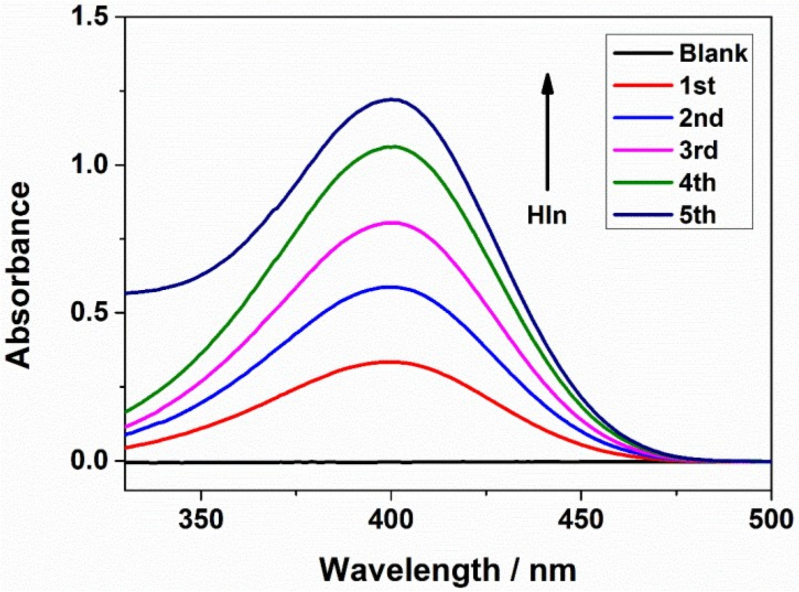

(a)

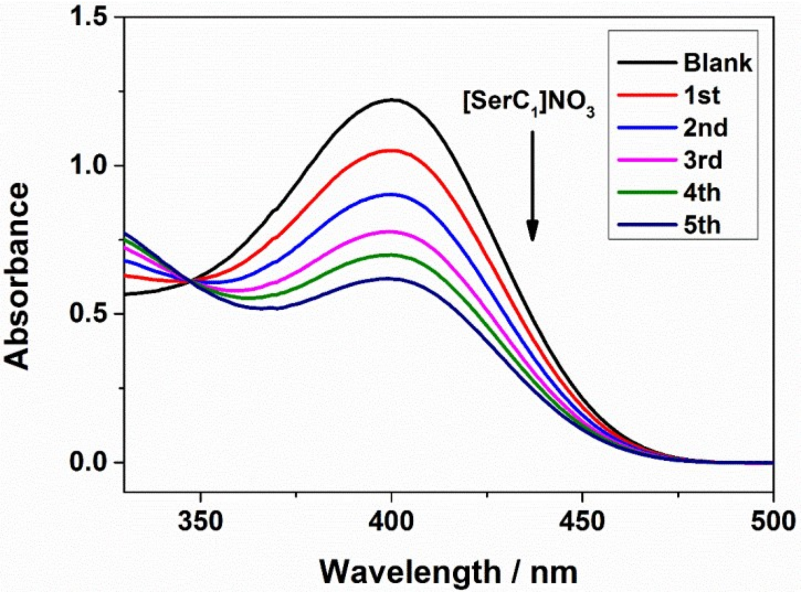

(b)

Figure S4. (**a**) The increasing absorbance during the deprotonation of the acid indicator (4-nitrophenol) by the base. (**b**) The decreasing absorbance of the acid indicator anion (4-nitrophenolate) during the titration of $[SerC_1]NO_3$ in water.

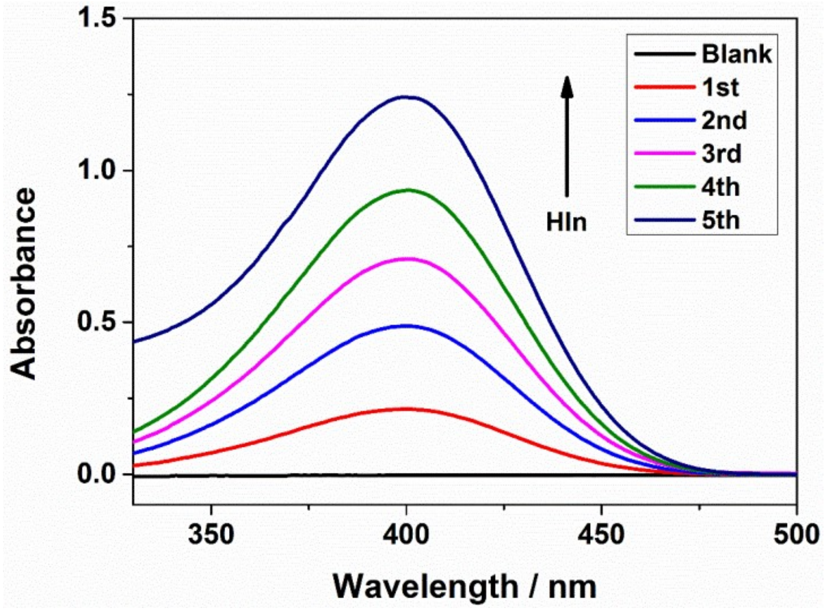

(a)

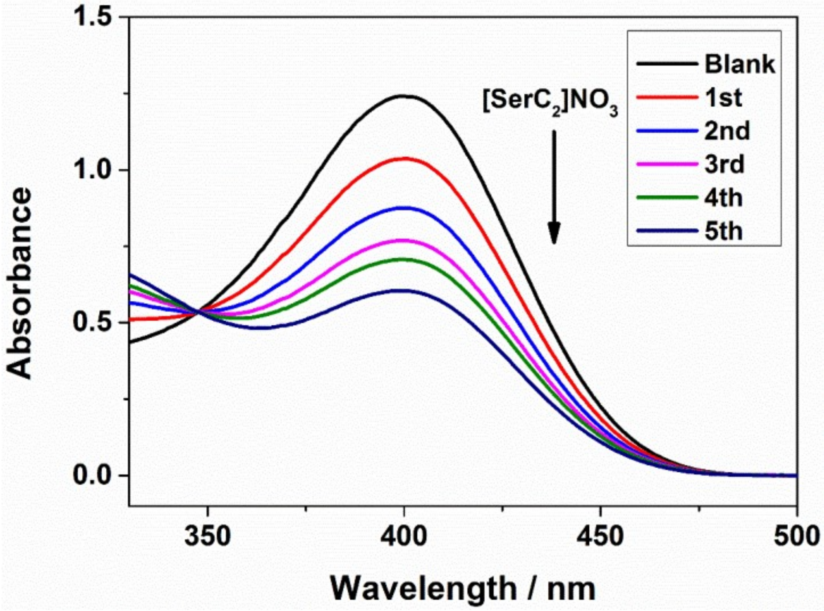

(b)

Figure S5. (**a**) The increasing absorbance during the deprotonation of the acid indicator (4-nitrophenol) by the base. (**b**) The decreasing absorbance of the acid indicator anion (4-nitrophenolate) during the titration of $[SerC_2]NO_3$ in water.

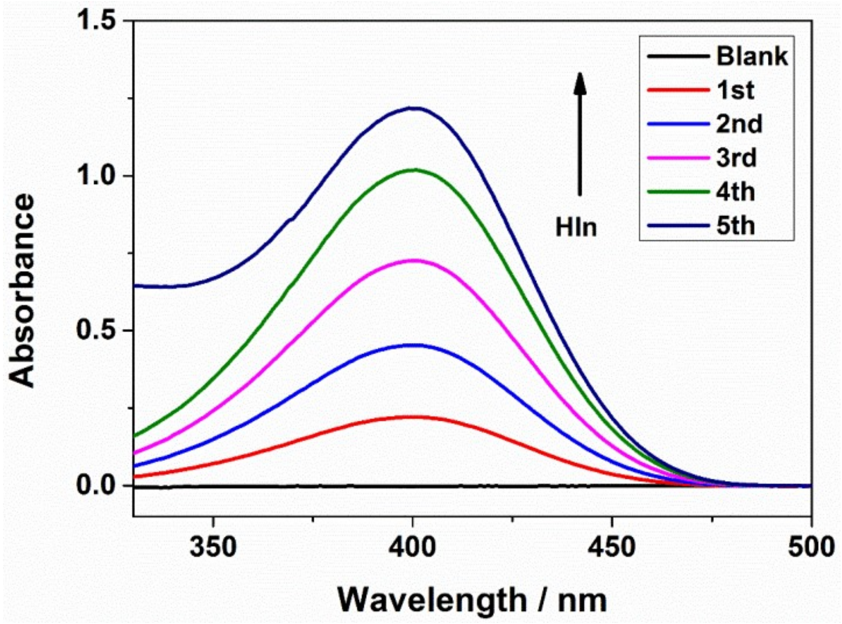

(a)

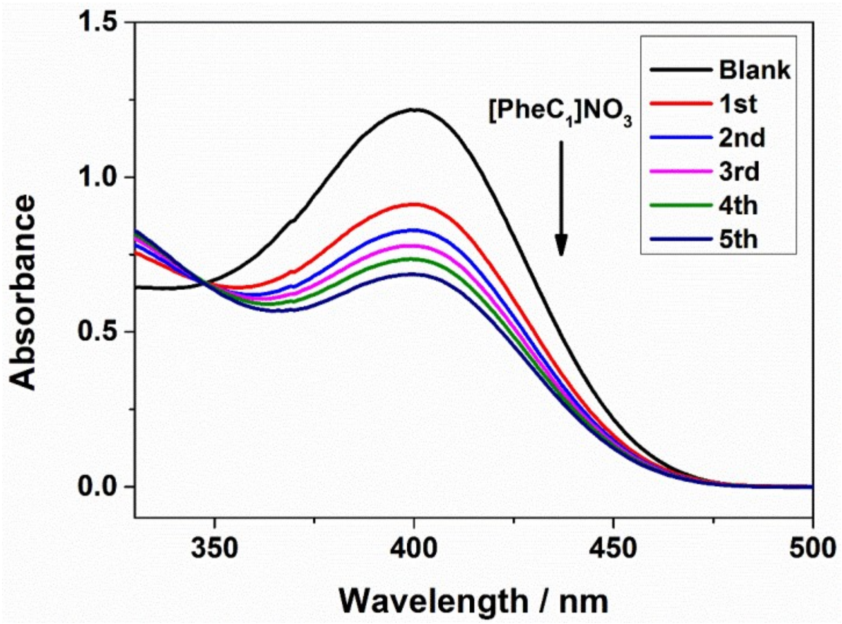

(b)

Figure S6. (**a**) The increasing absorbance during the deprotonation of the acid indicator (4-nitrophenol) by the base. (**b**) The decreasing absorbance of the acid indicator anion (4-nitrophenolate) during the titration of [PheC$_1$]NO$_3$ in water.

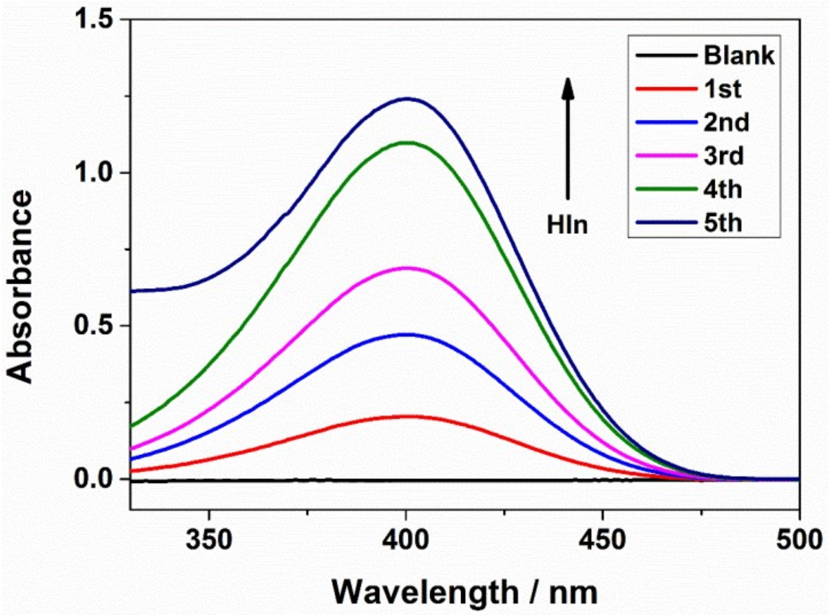

(a)

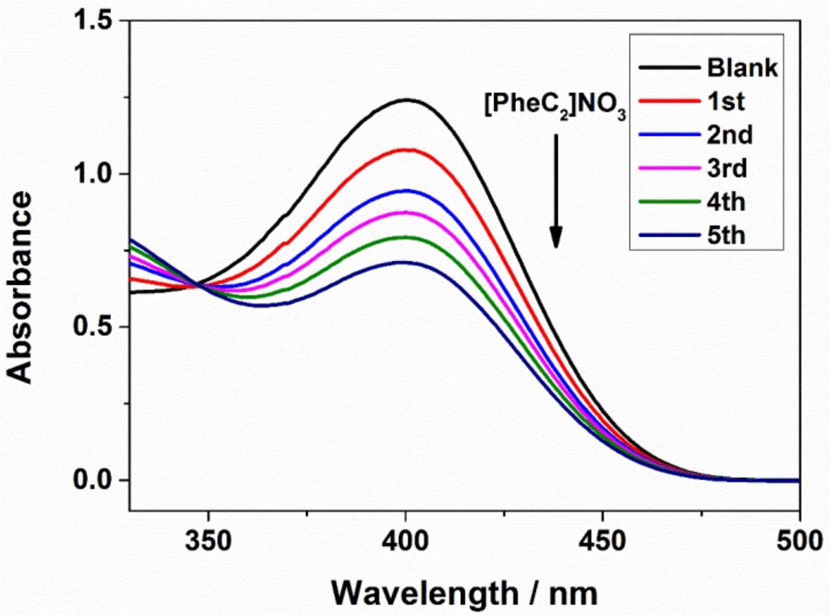

(b)

Figure S7. (**a**) The increasing absorbance during the deprotonation of the acid indicator (4-nitrophenol) by the base. (**b**) The decreasing absorbance of the acid indicator anion (4-nitrophenolate) during the titration of $[PheC_2]NO_3$ in water.

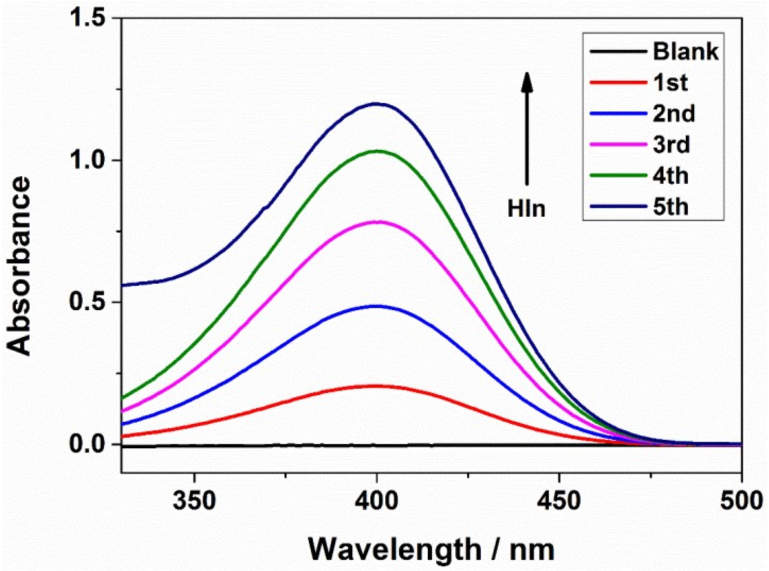

(a)

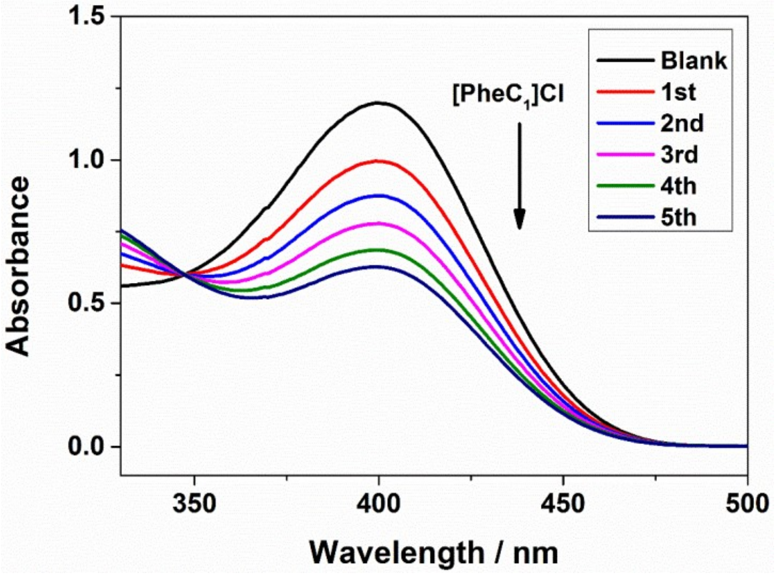

(b)

Figure S8. (**a**) The increasing absorbance during the deprotonation of the acid indicator (4-nitrophenol) by the base. (**b**) The decreasing absorbance of the acid indicator anion (4-nitrophenolate) during the titration of [PheC$_1$]Cl in water.

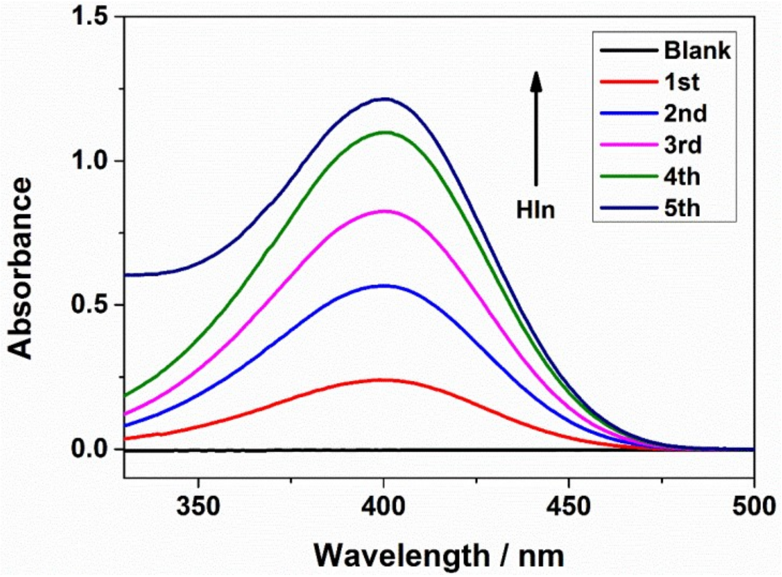

(a)

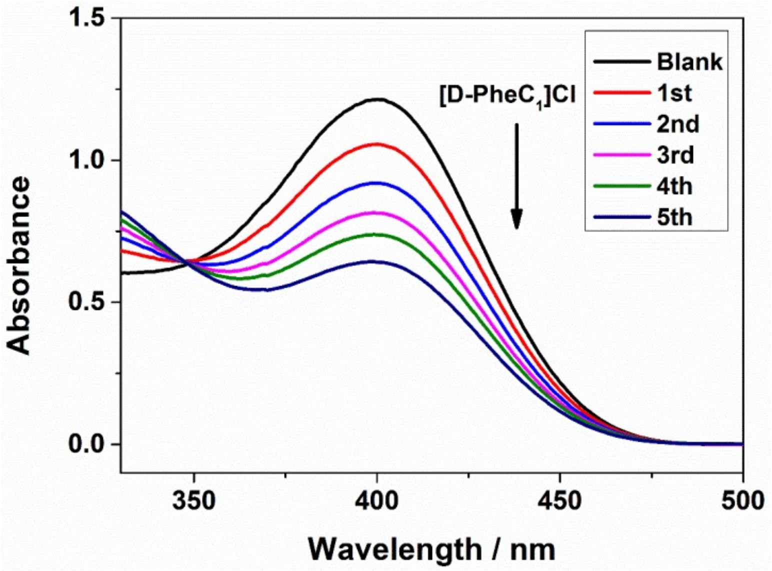

(b)

Figure S9. (**a**) The increasing absorbance during the deprotonation of the acid indicator (4-nitrophenol) by the base. (**b**) The decreasing absorbance of the acid indicator anion (4-nitrophenolate) during the titration of [D-PheC_1]Cl in water.

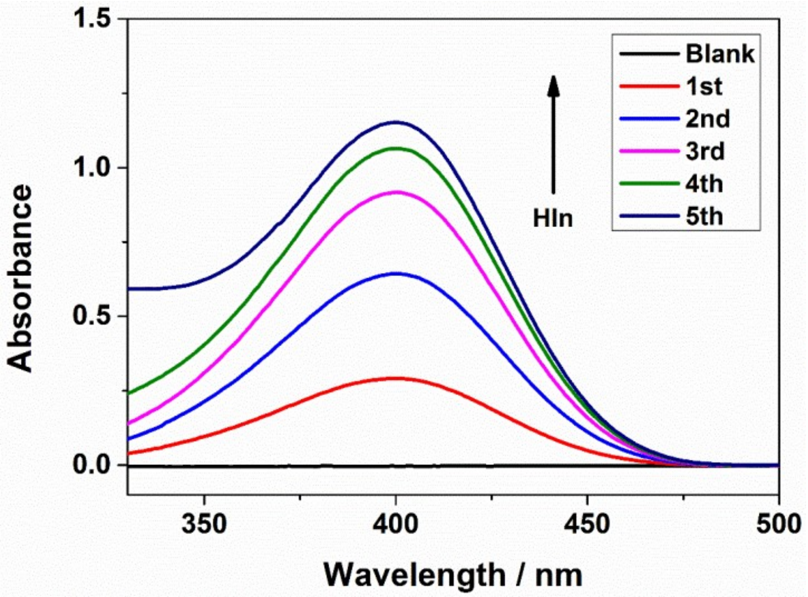

(a)

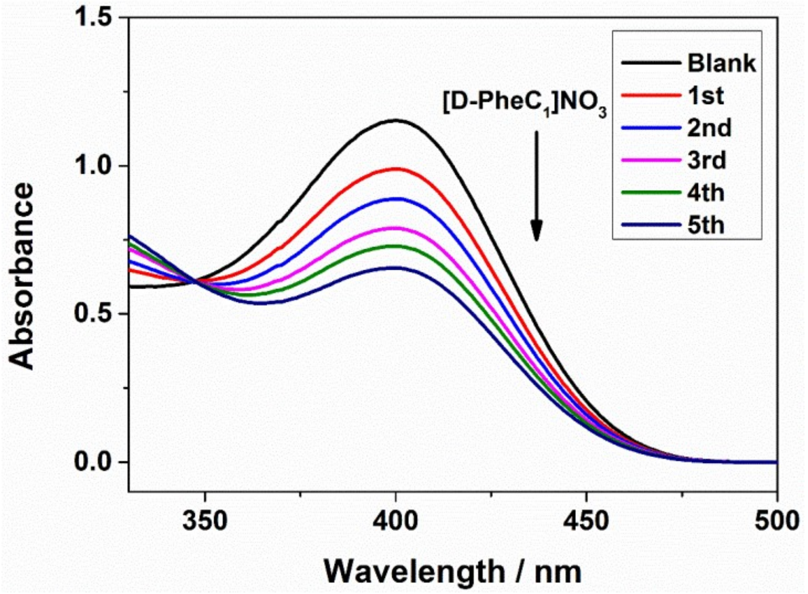

(b)

Figure S10. (**a**) The increasing absorbance during the deprotonation of the acid indicator (4-nitrophenol) by the base. (**b**) The decreasing absorbance of the acid indicator anion (4-nitrophenolate) during the titration of [D-PheC$_1$]NO$_3$ in water.

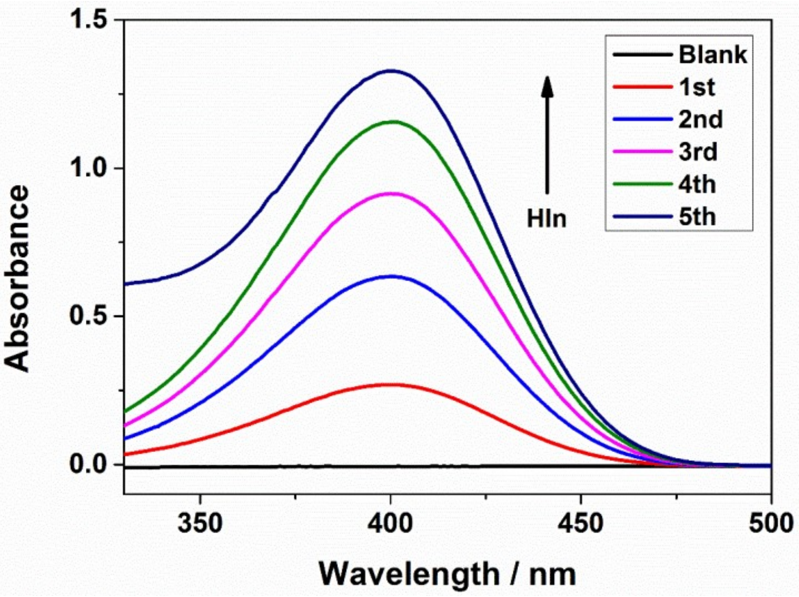

(a)

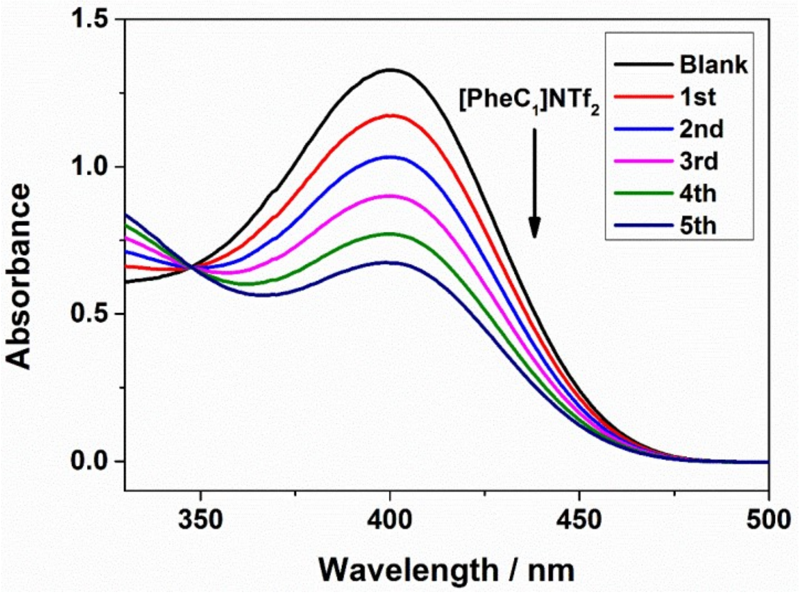

(b)

Figure S11. (**a**) The increasing absorbance during the deprotonation of the acid indicator (4-nitrophenol) by the base. (**b**) The decreasing absorbance of the acid indicator anion (4-nitrophenolate) during the titration of $[PheC_1]NTf_2$ in water.

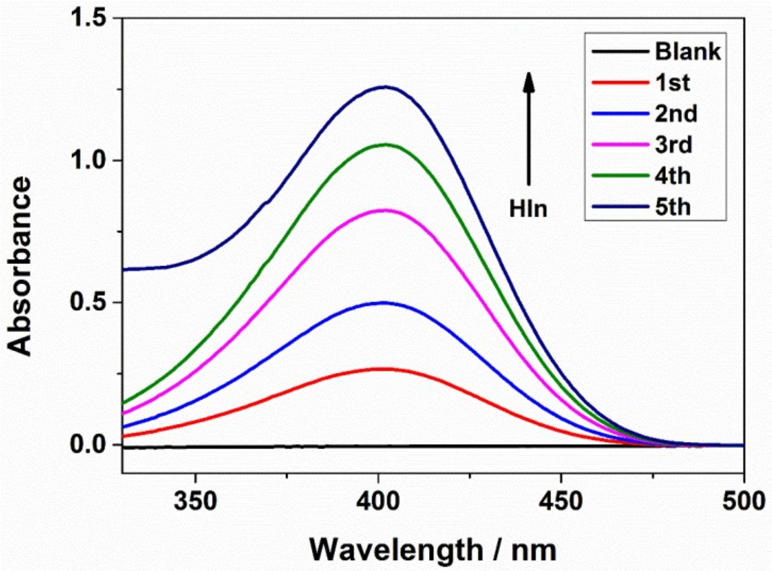

(a)

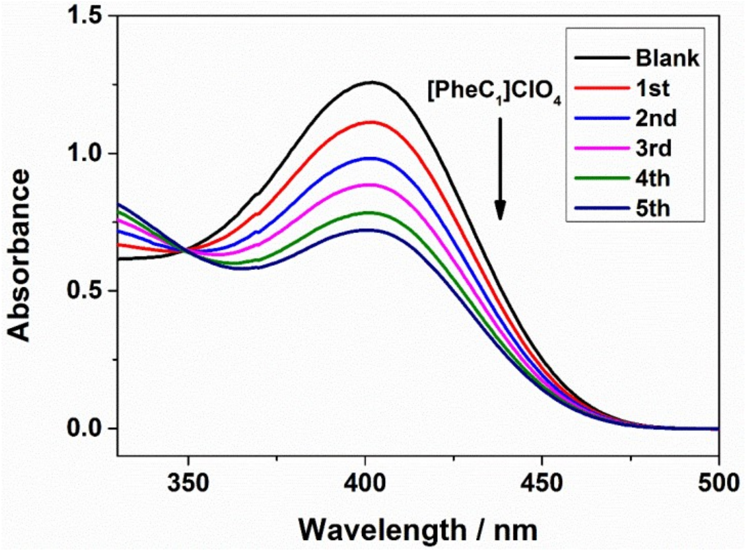

(b)

Figure S12. (**a**) The increasing absorbance during the deprotonation of the acid indicator (4-nitrophenol) by the base. (**b**) The decreasing absorbance of the acid indicator anion (4-nitrophenolate) during the titration of $[PheC_1]ClO_4$ in water.

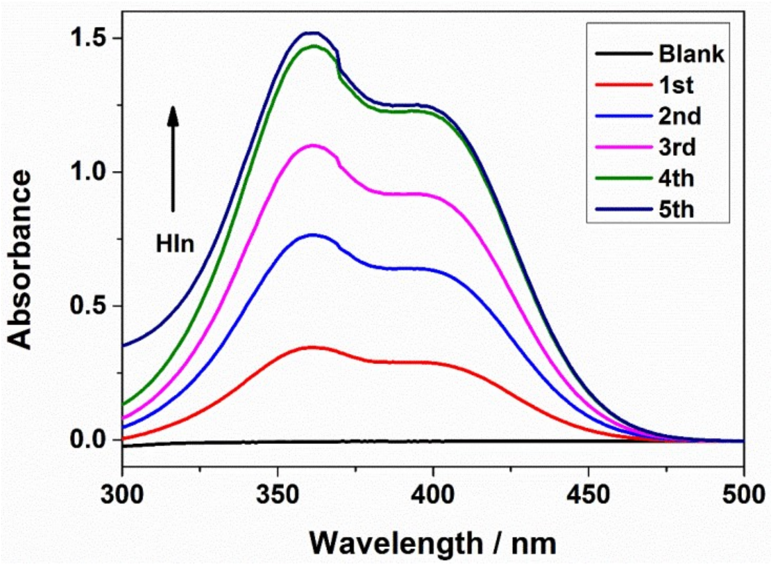

(a)

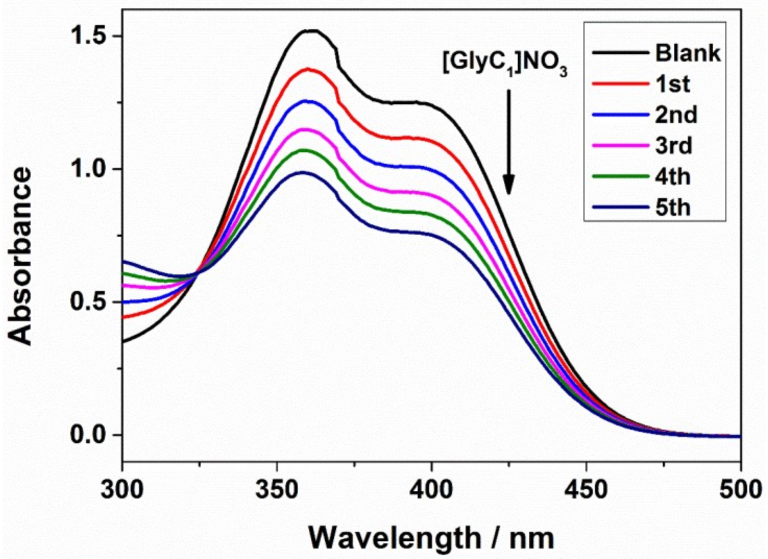

(b)

Figure S13. (**a**) The increasing absorbance during the deprotonation of the acid indicator (2,4-dinitrophenol) by the base. (**b**) The decreasing absorbance of the acid indicator anion (2,4-dinitrophenolate) during the titration of $[GlyC_1]NO_3$ in ethanol.

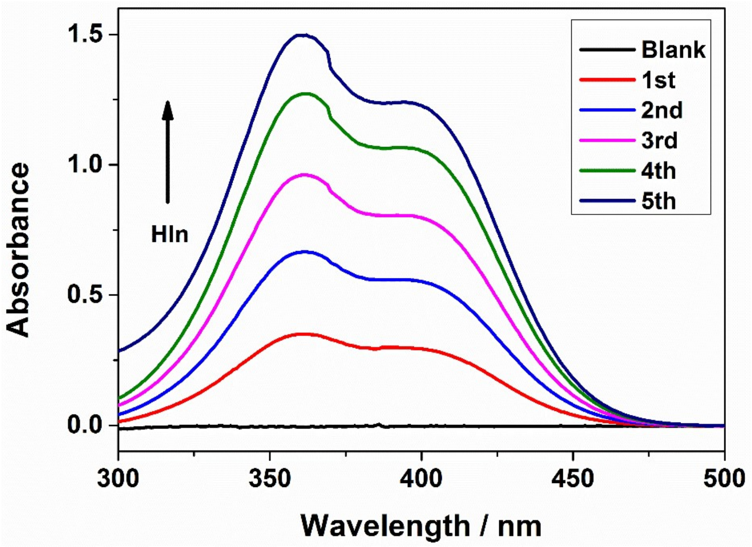

(a)

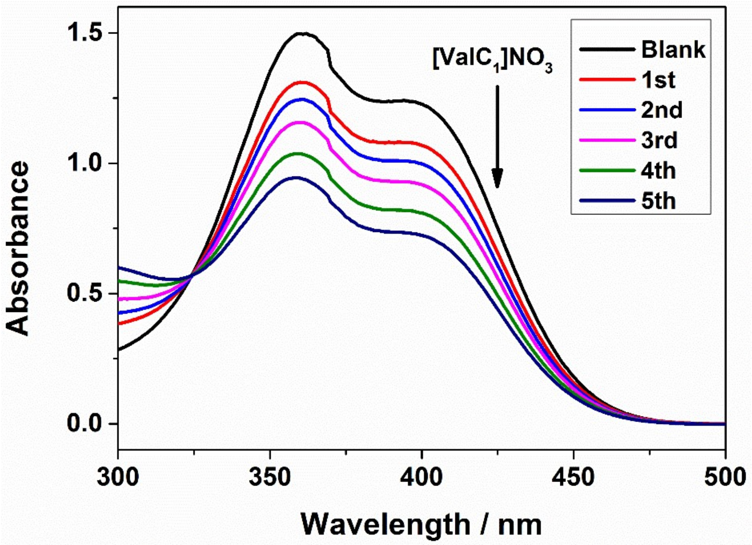

(b)

Figure S14. (**a**) The increasing absorbance during the deprotonation of the acid indicator (2,4-dinitrophenol) by the base. (**b**) The decreasing absorbance of the acid indicator anion (2,4-dinitrophenolate) during the titration of $[ValC_1]NO_3$ in ethanol.

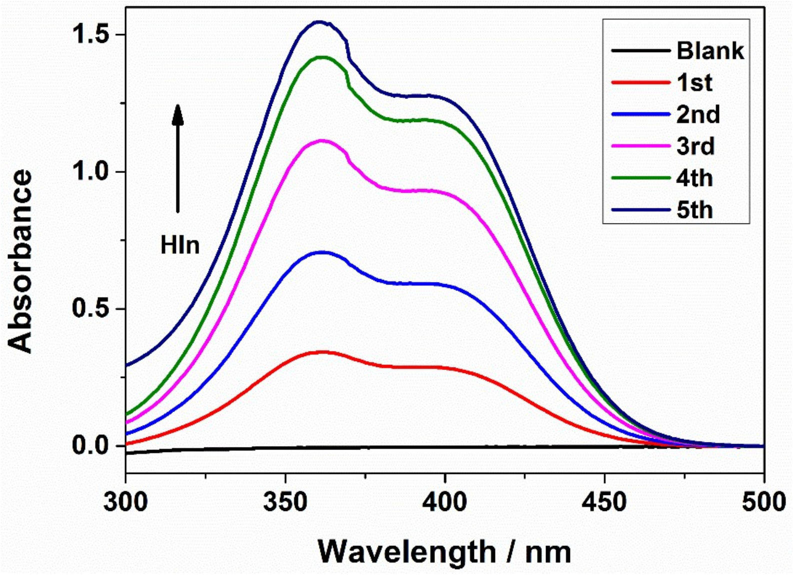

(a)

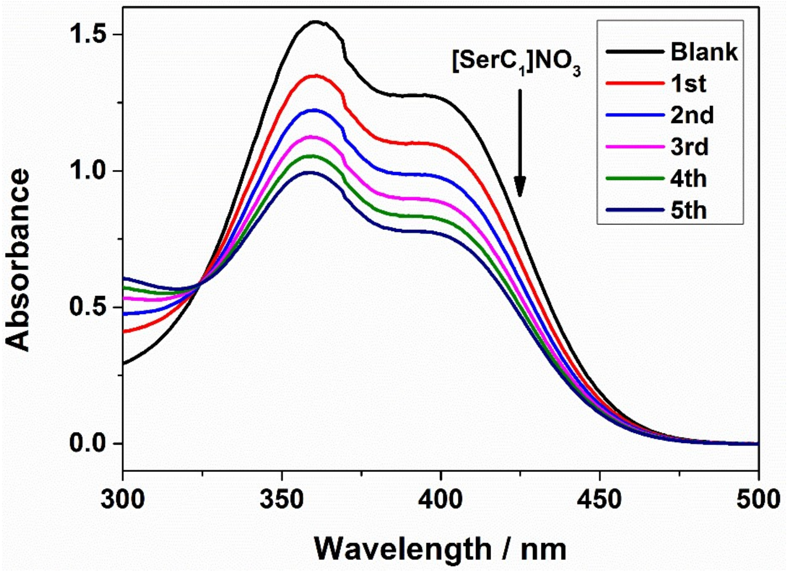

(b)

Figure S15. (**a**) The increasing absorbance during the deprotonation of the acid indicator (2,4-dinitrophenol) by the base. (**b**) The decreasing absorbance of the acid indicator anion (2,4-dinitrophenolate) during the titration of $[SerC_1]NO_3$ in ethanol.

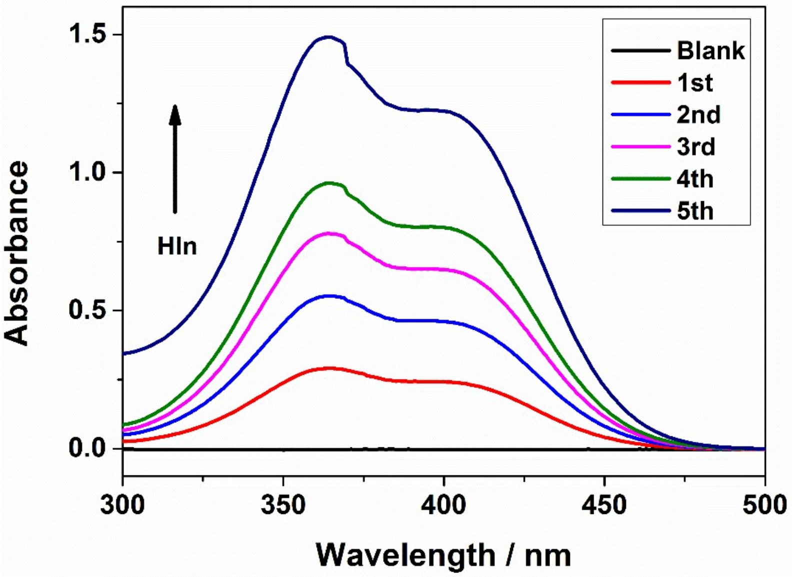

(a)

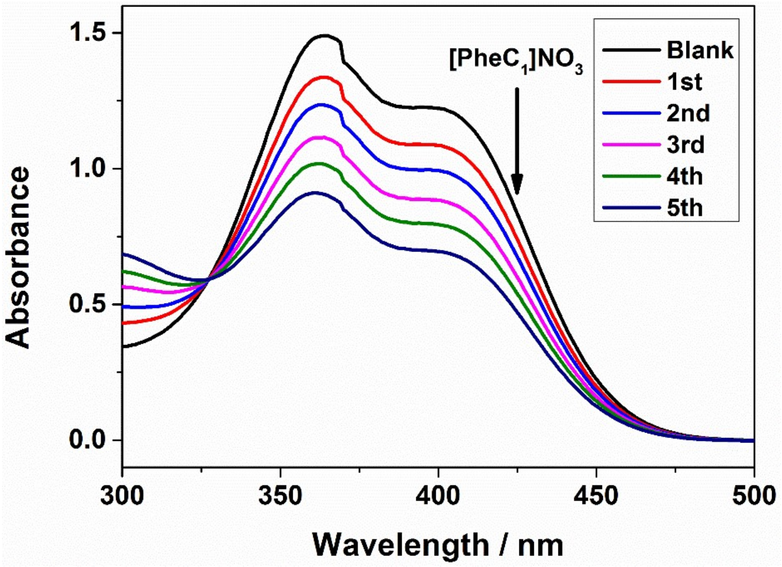

(b)

Figure S16. (**a**) The increasing absorbance during the deprotonation of the acid indicator (2,4-dinitrophenol) by the base. (**b**) The decreasing absorbance of the acid indicator anion (2,4-dinitrophenolate) during the titration of $[PheC_1]NO_3$ in ethanol.

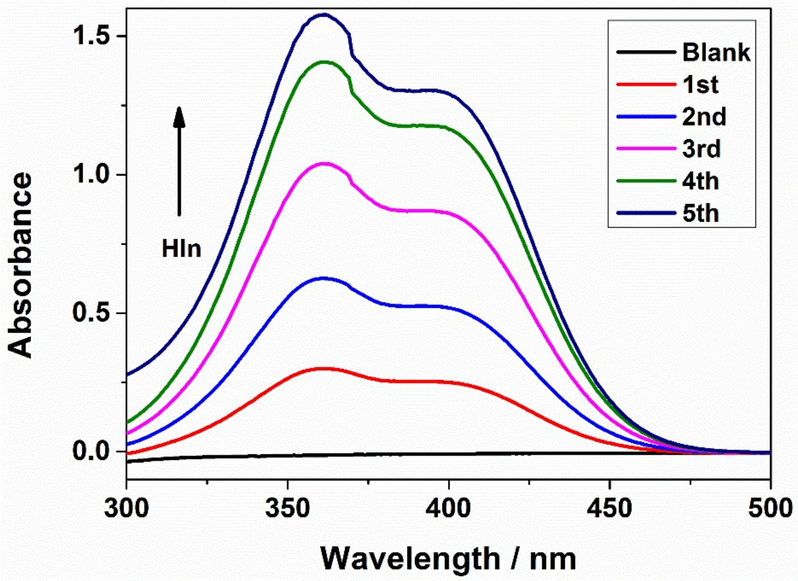

(a)

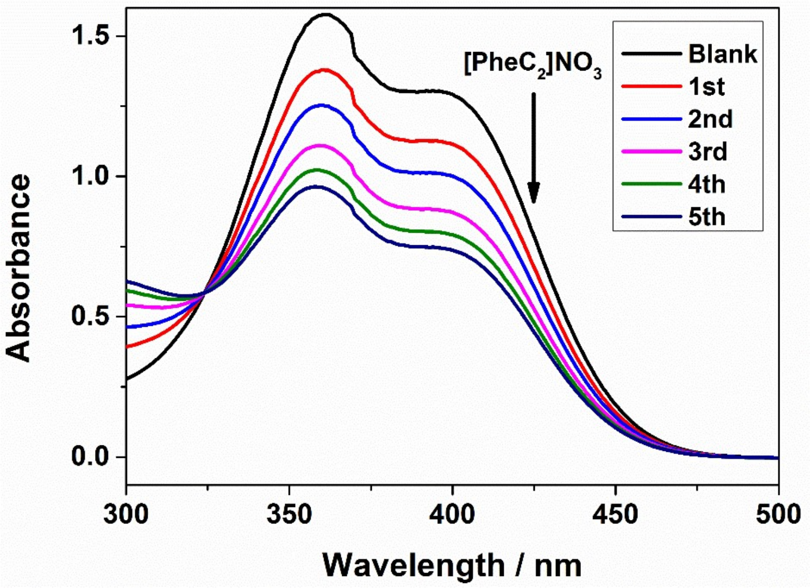

(b)

Figure S17. (**a**) The increasing absorbance during the deprotonation of the acid indicator (2,4-dinitrophenol) by the base. (**b**) The decreasing absorbance of the acid indicator anion (2,4-dinitrophenolate) during the titration of $[PheC_2]NO_3$ in ethanol.

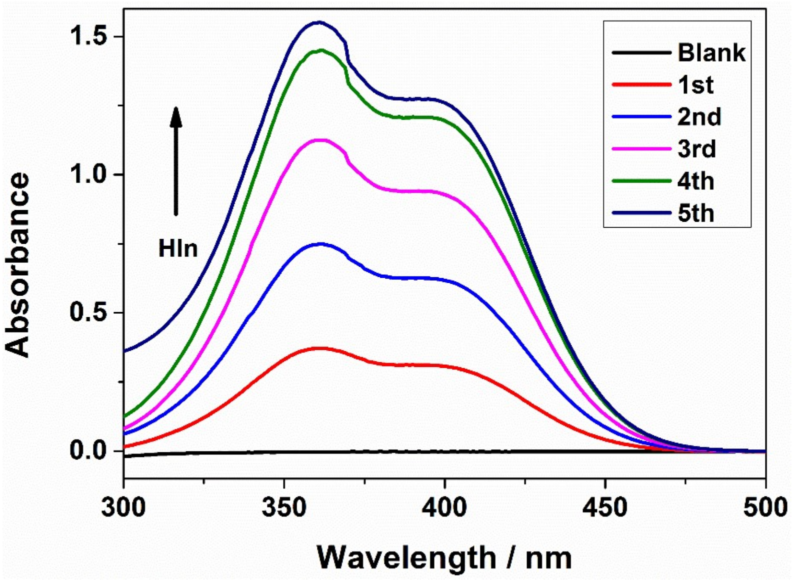

(a)

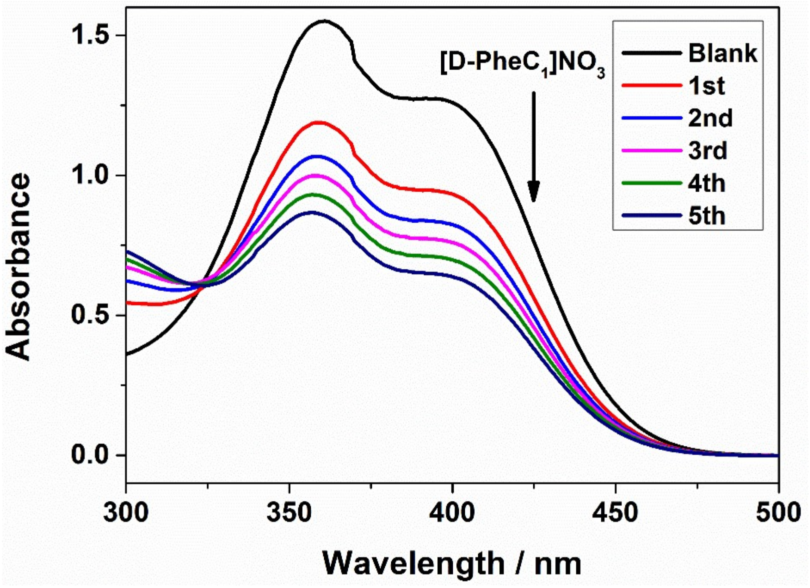

(b)

Figure S18. (**a**) The increasing absorbance during the deprotonation of the acid indicator (2,4-dinitrophenol) by the base. (**b**) The decreasing absorbance of the acid indicator anion (2,4-dinitrophenolate) during the titration of [D-PheC$_1$]NO_3 in ethanol.

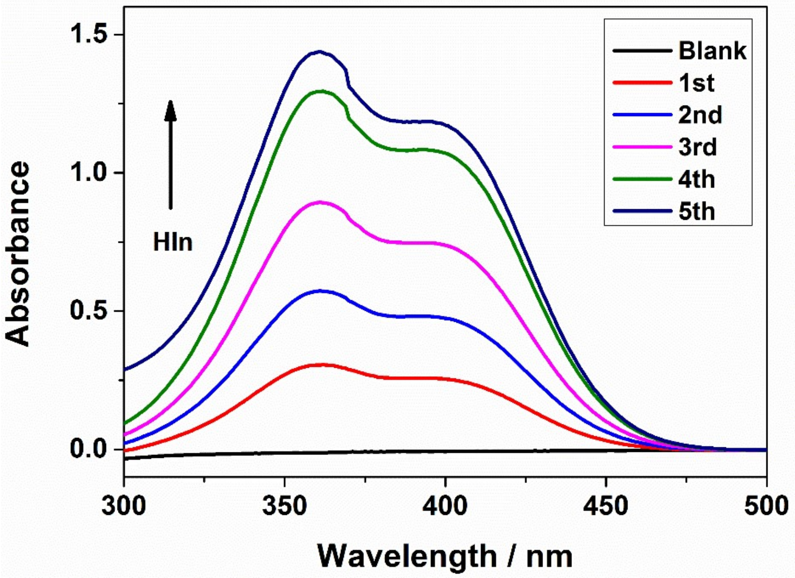

(a)

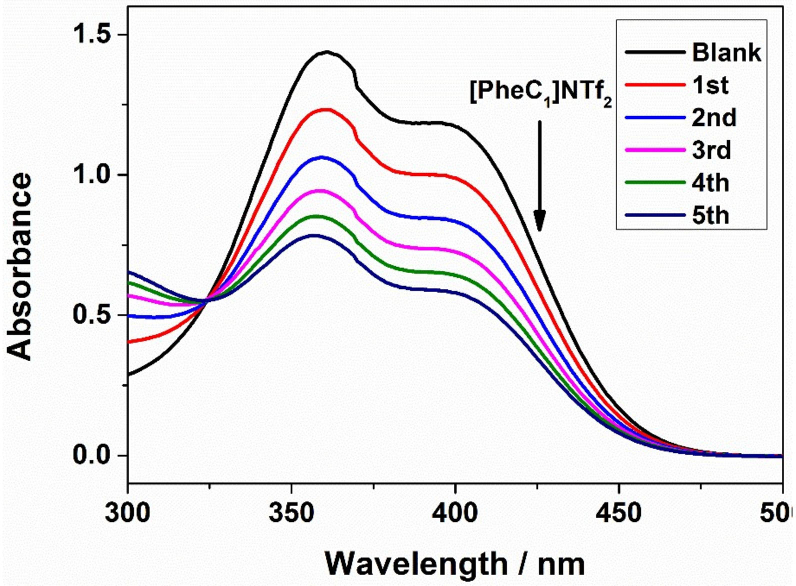

(b)

Figure S18. (**a**) The increasing absorbance during the deprotonation of the acid indicator (2,4-dinitrophenol) by the base. (**b**) The decreasing absorbance of the acid indicator anion (2,4-dinitrophenolate) during the titration of [PheC$_1$]NTf$_2$ in ethanol.

The optimized geometry coordinates of [AAE]X.

The optimized geometry coordinates (Å) (B3LYP/6-311++G(d,p) level)

[GlyC$_1$]$^+$

C	1.15736700	−0.83142200	0.00022400
C	−0.10147200	0.04680400	−0.00003500
H	1.19788600	−1.46272100	−0.88621800
H	1.19763300	−1.46182100	0.88735300
O	0.00377400	1.25482500	0.00009800

O	−1.19023800	−0.66888800	−0.00022700
C	−2.47414900	0.04265500	0.00001700
H	−2.54187600	0.65975000	−0.89390700
H	−3.22221400	−0.74331900	−0.00314400
H	−2.54414600	0.65451200	0.89738300
H	2.91084300	0.04703500	−0.82808400
H	1.86825200	1.08090800	0.00039800
H	2.91179600	0.04656400	0.82706400
N	2.31758000	0.13476700	−0.00015000

GlyC1

C	1.24658600	−0.72955800	0.00038000
C	−0.03365700	0.10293100	0.00009800
H	1.21224100	−1.39103500	−0.87073400
H	1.21259400	−1.38962700	0.87259100
O	−0.08246500	1.30842400	0.00021300
O	−1.12257400	−0.69246100	−0.00021000
C	−2.40014400	−0.02589400	−0.00009600
H	−2.50442400	0.59686300	−0.88955000
H	−3.14254700	−0.82074100	0.00042800
H	−2.50392300	0.59762500	0.88887500
N	2.48750400	0.02015800	−0.00038600
H	2.53824600	0.62634800	−0.81256700
H	2.53888900	0.62688700	0.81134800

[GlyC2]+

C	−1.57639400	−0.89895400	−0.00013100
C	−0.44553900	0.14167700	−0.00015700
H	−1.53222300	−1.52931300	0.88669100
H	−1.53258700	−1.52906100	−0.88714400
O	−0.72066900	1.32438500	0.00029800
O	0.72837100	−0.41663100	−0.00070400
C	1.92543200	0.47082900	−0.00061100
H	1.85288300	1.09740500	−0.88968100
H	1.85182100	1.09886700	0.88732600
C	3.14412800	−0.41756600	0.00078300
H	3.17699200	−1.05033600	−0.88771200
H	4.03574400	0.21421500	0.00046900
H	3.17632300	−1.04851800	0.89059500
H	−3.43129400	−0.26064000	0.82810400
H	−2.52933300	0.90115000	0.00034500
H	−3.43178600	−0.26048600	−0.82718200
N	−2.85484600	−0.09589000	0.00030500

GlyC2

C	1.65807200	−0.81825600	0.00006500
C	0.52134000	0.20271700	0.00004500
H	1.52228200	−1.46593300	−0.87157100
H	1.52243200	−1.46567400	0.87191900
O	0.66442700	1.40133800	0.00013200
O	−0.67644600	−0.41361000	−0.00009200
C	−1.85188000	0.43786800	−0.00016700

H	−1.81493400	1.08032300	0.88247600
H	−1.81508200	1.07997700	−0.88307100
C	−3.07030300	−0.46171400	0.00010200
H	−3.08662100	−1.09980200	0.88645600
H	−3.97742900	0.14859000	0.00005800
H	−3.08676000	−1.10013700	−0.88600700
N	3.00029600	−0.26966700	−0.00010300
H	3.14327600	0.32249900	−0.81171200
H	3.14354600	0.32231500	0.81159000

$[ValC_1]^+$

C	0.56236600	0.50174100	−0.70076200
H	0.49897500	0.33956000	−1.77738100
C	1.60779700	−0.46365000	−0.08290500
C	−0.84980900	0.44417600	−0.08763200
H	2.57436600	−0.12383500	−0.48102400
C	1.65258400	−0.38560300	1.45020300
C	1.40209100	−1.90083700	−0.58145400
O	−1.27782600	1.37372000	0.56585700
H	0.72877400	−0.76200500	1.89750100
H	2.47226300	−1.00085900	1.82447900
H	1.81806900	0.62739500	1.83323600
H	1.39189500	−1.95335600	−1.67299700
H	2.22489000	−2.52420000	−0.22694500
H	0.47029800	−2.32850800	−0.21002600
O	−1.47438800	−0.67196500	−0.36495600
C	−2.83304200	−0.82869500	0.16065800
H	−3.15306600	−1.80501200	−0.18915700
H	−2.80956800	−0.78501200	1.24804700
H	−3.46665900	−0.03839100	−0.23794600
H	1.91699600	2.02501400	−0.07273500
H	0.95712500	2.50406000	−1.33672400
H	0.27484000	2.34208000	0.18491200
N	0.98094100	1.95231900	−0.47701300

$ValC_1$

C	−0.53379900	−0.51826400	−0.74803900
H	−0.30911400	−0.24054500	−1.78065600
C	−1.58545700	0.50117100	−0.21998200
C	0.77786200	−0.44429300	0.04000800
H	−2.45020500	0.33540500	−0.87174100
C	−2.02778300	0.22050300	1.22245700
C	−1.13140000	1.95712400	−0.38928500
O	1.01545600	−1.07629300	1.04262200
H	−1.20254900	0.33659600	1.93067100
H	−2.81778500	0.91915000	1.51200800
H	−2.43031600	−0.78896000	1.33349700
H	−0.81754600	2.16579200	−1.41592500
H	−1.95291300	2.63619100	−0.14501900
H	−0.29374700	2.20379100	0.26938000
O	1.65739200	0.41346300	−0.51861100
C	2.91577700	0.56938500	0.16686600

H	3.48520700	1.27923500	−0.42892000
H	2.75574200	0.95441600	1.17488900
H	3.43596600	−0.38729800	0.22803400
N	−1.09519600	−1.86904300	−0.75517000
H	−0.52781300	−2.48531800	−1.32820200
H	−1.08253700	−2.25627100	0.18393700

[SerC1]+

C	0.73480600	0.24302800	−0.71796600
H	0.64626000	0.43149000	−1.78951200
C	1.41694600	−1.10223600	−0.45731900
C	−0.66097300	0.40678500	−0.08103100
H	2.28294000	−1.22359100	−1.11787400
O	−0.93266900	1.39548000	0.56139500
O	−1.44791800	−0.59944000	−0.36862800
C	−2.82363800	−0.53845500	0.13215000
H	−3.28784600	−1.45318100	−0.22235100
H	−2.80887200	−0.49633900	1.21969200
H	−3.31702000	0.34095800	−0.27787800
O	1.81019700	−1.04972200	0.90889600
H	2.20864600	−1.87930000	1.19484400
H	0.70014900	−1.90116400	−0.65821300
H	2.09141000	0.92488700	0.68806700
H	2.20739100	1.79665600	−0.74401500
H	0.89230700	2.03000400	0.29961800
N	1.56355700	1.34347200	−0.09438500

SerC1

C	0.80434900	0.31321800	−0.67601300
H	0.66722500	0.43239200	−1.76092800
C	1.41418700	−1.07953100	−0.42086700
C	−0.58609900	0.45178000	−0.06046100
H	2.33922000	−1.15942900	−1.01022500
O	−0.92534600	1.35568400	0.66505900
O	−1.40010400	−0.53799900	−0.45556200
C	−2.74300200	−0.50394700	0.06881900
H	−3.24243500	−1.36919900	−0.36051000
H	−2.72353700	−0.57104700	1.15687800
H	−3.24305600	0.41839600	−0.22894600
N	1.68570400	1.32618100	−0.09283900
H	2.31995400	1.71725200	−0.77792600
H	1.14180600	2.07491100	0.32430700
O	1.67146400	−1.26734000	0.95644800
H	2.10439900	−0.45187200	1.25198000
H	0.73177600	−1.86655300	−0.74117300

[SerC2]+

C	1.18237500	−0.12482900	0.73197000
H	1.09296800	−0.27719400	1.80908000
C	1.52834900	1.33181900	0.41671500
C	−0.10312500	−0.65641500	0.05954100
H	2.30645800	1.69446700	1.09803500

O	−0.08681600	−1.71063700	−0.53764100
O	−1.12419500	0.13127000	0.26695800
C	−2.44044200	−0.29040900	−0.27885500
H	−2.32508800	−0.36529300	−1.36037200
H	−2.65042700	−1.28078400	0.12550400
O	1.98695600	1.31305100	−0.93001300
H	2.18952800	2.20044300	−1.24634900
H	0.62972800	1.93857200	0.54639700
H	2.73952100	−0.51992400	−0.57586500
H	2.97115500	−1.29014900	0.90017300
H	1.80111200	−1.85567500	−0.19117300
N	2.27888100	−1.01993700	0.20075600
C	−3.45634500	0.74487100	0.13640100
H	−4.43364800	0.45313700	−0.25571600
H	−3.53470500	0.81321000	1.22289500
H	−3.21119700	1.72904700	−0.26696400

SerC$_2$

C	−1.25839800	−0.16480700	−0.70158400
H	−1.09617900	−0.25251400	−1.78614300
C	−1.51327500	1.31923700	−0.37391600
C	0.01781800	−0.68818700	−0.04331700
H	−2.35586400	1.66521300	−0.99027900
O	0.07523900	−1.69638700	0.62035700
O	1.07186500	0.08907100	−0.32375400
C	2.34611000	−0.30789200	0.25107900
H	2.23307200	−0.35238900	1.33604100
H	2.58765400	−1.31180600	−0.10499900
O	−1.78972200	1.48605800	1.00272200
H	−2.42602600	0.78891600	1.22398500
H	−0.63944000	1.92505300	−0.61279700
C	3.37833900	0.71480500	−0.17434600
H	4.35260000	0.44535900	0.24205500
H	3.46984100	0.75273100	−1.26217600
H	3.11502000	1.71094800	0.18759300
N	−2.39618600	−0.95316800	−0.22475100
H	−3.07030300	−1.12915200	−0.95908500
H	−2.07969500	−1.83905500	0.15696000

[PheC$_1$]$^+$

C	−1.10651900	1.31199800	−0.35063800
H	−1.78934500	2.02170300	−0.82305500
C	−0.03682600	0.84524700	−1.36678800
C	−1.96897800	0.20634400	0.28481600
H	0.30500200	1.71860700	−1.92830800
O	−2.11792900	0.14388200	1.48582000
O	−2.51703200	−0.57034000	−0.61896000
C	−3.43051200	−1.60894400	−0.14130000
H	−3.75316400	−2.12859000	−1.03800400
H	−2.90038200	−2.27650900	0.53573700
H	−4.27179300	−1.14268500	0.36879100
C	1.14105800	0.15570900	−0.71064800

C	2.30781600	0.88163600	−0.42631300
C	1.08305900	−1.19628200	−0.34751100
C	3.38789200	0.27230900	0.21587200
H	2.39818900	1.91372100	−0.75571300
C	2.16204600	−1.80365900	0.28949100
H	0.20384300	−1.78447300	−0.58803000
C	3.31324200	−1.06987500	0.57771300
H	4.28878900	0.84128900	0.41277200
H	2.10969000	−2.85369700	0.55184700
H	4.15301500	−1.54794000	1.06751900
H	−0.54283000	0.19034600	−2.07794400
H	−0.49758700	3.02886200	0.76863400
H	−0.98066500	1.68362200	1.67507600
H	0.51654100	1.70527900	0.91655900
N	−0.46761400	2.01074600	0.83301000

PheC$_1$

C	−1.20304400	1.36616800	−0.35621500
H	−1.97766600	1.83829600	−0.97112400
C	−0.09160700	0.90416800	−1.33669500
C	−1.89115500	0.18048500	0.33784700
H	0.24390100	1.79997000	−1.86420000
O	−1.96932100	0.02511200	1.53120500
O	−2.44177800	−0.66670200	−0.55932300
C	−3.14960300	−1.79649600	−0.01231000
H	−3.52117100	−2.35112300	−0.87119900
H	−2.47732500	−2.41440300	0.58442500
H	−3.97630700	−1.45975400	0.61457900
C	1.09224600	0.22766100	−0.67901700
C	2.17999700	0.97711500	−0.21292600
C	1.12848400	−1.16322600	−0.52034900
C	3.26554600	0.35631800	0.40300200
H	2.17577300	2.05430700	−0.34092600
C	2.21251100	−1.78712000	0.09396000
H	0.30474600	−1.76322400	−0.89332200
C	3.28435600	−1.02833700	0.56073500
H	4.09925300	0.95440800	0.75430200
H	2.22275200	−2.86614500	0.20250800
H	4.12995600	−1.51243700	1.03619800
H	−0.54031900	0.23347200	−2.07188300
N	−0.69832700	2.36370400	0.57334900
H	−1.45055000	2.71062900	1.15965100
H	−0.02235000	1.94237800	1.20429000

[PheC$_2$*]*$^+$

C	0.54226500	1.55587300	0.44517800
H	1.12675800	2.29967800	0.99111600
C	−0.33222400	0.74054900	1.42753600
C	1.53037800	0.74726900	−0.41844800
H	−0.76338700	1.43069800	2.15724900
O	1.57520600	0.90921900	−1.62026500
O	2.28634600	−0.04050000	0.30280300

C	3.34450000	−0.80690200	−0.39920900
H	2.85035800	−1.42451100	−1.14971400
H	3.98203000	−0.08128900	−0.90511500
C	4.08305900	−1.61640400	0.63869600
H	3.41997300	−2.32430300	1.13940600
H	4.87364500	−2.18522800	0.14335700
H	4.54743300	−0.97319700	1.38823900
C	−1.43246900	−0.03511100	0.73354000
C	−2.72797200	0.49912700	0.66125100
C	−1.17727300	−1.26857900	0.11971600
C	−3.74253900	−0.17943000	−0.01731800
H	−2.96254800	1.42444500	1.18137600
C	−2.19049900	−1.94586400	−0.55388200
H	−0.19072100	−1.71434800	0.18919500
C	−3.47257000	−1.40051500	−0.62905100
H	−4.74200300	0.23791100	−0.04956400
H	−1.98271000	−2.90521900	−1.01287700
H	−4.25940200	−1.93328700	−1.14938500
H	0.34204400	0.08072000	1.97587000
H	−0.44361800	3.28283400	−0.34219600
H	0.17474500	2.18865000	−1.47836900
H	−1.23418800	1.83571700	−0.63312000
N	−0.31125300	2.29584300	−0.56483200

PheC$_2$

C	−0.57695700	1.68672300	−0.41564500
H	−1.28352200	2.23918600	−1.04532600
C	0.32963800	0.88011700	−1.38363200
C	−1.43801600	0.77039200	0.46891400
H	0.79991600	1.61114800	−2.04528500
O	−1.43446700	0.77317300	1.67544600
O	−2.23750500	−0.02460600	−0.27335100
C	−3.13204100	−0.90697000	0.45294800
H	−2.53206100	−1.56947600	1.08097900
H	−3.75794200	−0.30074800	1.11162300
C	−3.95146600	−1.67261600	−0.56533600
H	−3.31087300	−2.27036500	−1.21774300
H	−4.63964400	−2.34776600	−0.04968000
H	−4.53942600	−0.99330600	−1.18655900
C	1.39714600	0.04811100	−0.70511000
C	2.65280500	0.59363400	−0.40911100
C	1.15398800	−1.28576400	−0.35446700
C	3.63166200	−0.16736400	0.22814700
H	2.86328100	1.62104600	−0.68635300
C	2.13053400	−2.04970300	0.28140900
H	0.19364400	−1.73174000	−0.59184100
C	3.37278300	−1.49146100	0.57761600
H	4.59843400	0.27298100	0.44636500
H	1.92338200	−3.08211100	0.54109000
H	4.13454400	−2.08527300	1.07016800
H	−0.30773300	0.23898400	−1.99566100
N	0.20054500	2.65433700	0.34196300

H	−0.41187800	3.22447100	0.91634700
H	0.82138400	2.17347800	0.98698200

[D−PheC1]+

C	1.10637500	1.31205900	−0.35016500
H	1.78897100	2.02209800	−0.82237200
C	0.03677300	0.84545300	−1.36660900
C	1.96907600	0.20635700	0.28494200
H	−0.30477800	1.71898500	−1.92803300
H	0.54277100	0.19057600	−2.07776400
C	−1.14125200	0.15588200	−0.71077600
O	2.11769600	0.14329000	1.48595400
C	−2.30814400	0.88148200	−0.42681000
C	−1.08287100	−1.19613000	−0.34729200
H	−2.39884700	1.91350000	−0.75632600
C	−3.38824300	0.27192600	0.21539700
C	−2.16169800	−1.80362400	0.28965300
H	−0.20347300	−1.78408500	−0.58772400
H	−4.28932700	0.84073500	0.41195300
C	−3.31324200	−1.07005200	0.57755100
H	−2.10925700	−2.85358200	0.55234600
H	−4.15287200	−1.54835400	1.06737600
O	2.51750200	−0.56972700	−0.61918100
C	3.43122200	−1.60826300	−0.14191300
H	3.74854200	−2.13207000	−1.03808900
H	4.27595100	−1.14144400	0.36194800
H	2.90330700	−2.27201700	0.54053000
H	0.49617600	3.02831000	0.76940700
H	−0.51688900	1.70392500	0.91721700
H	0.98028800	1.68350100	1.67581700
N	0.46712500	2.01012500	0.83366600

D−PheC1

C	1.20273000	1.36638400	−0.35574200
H	1.97738600	1.83896600	−0.97028200
C	0.09155300	0.90452300	−1.33655000
C	1.89098800	0.18057700	0.33791000
H	−0.24400200	1.80038900	−1.86391400
H	0.54046900	0.23404200	−2.07180500
C	−1.09226500	0.22776900	−0.67908000
O	1.96914300	0.02476700	1.53119500
C	−2.18046400	0.97686300	−0.21351300
C	−1.12783600	−1.16308600	−0.51988500
H	−2.17672100	2.05401600	−0.34184200
C	−3.26587900	0.35574700	0.40237400
C	−2.21170600	−1.78728900	0.09433600
H	−0.30358100	−1.76266900	−0.89236700
H	−4.09993800	0.95358600	0.75326300
C	−3.28407000	−1.02884500	0.56053700
H	−2.22147600	−2.86627300	0.20329800
H	−4.12955400	−1.51323000	1.03591500
O	2.44181500	−0.66618800	−0.55958600

C	3.15011200	−1.79588400	−0.01298000
H	3.52285300	−2.34943700	−0.87205700
H	3.97598300	−1.45907400	0.61496900
H	2.47781300	−2.41489700	0.58260200
N	0.69758300	2.36336800	0.57419000
H	0.02145900	1.94166600	1.20472400
H	1.44958700	2.71015600	1.16085500

H_3O^+

O	0.00000300	0.00002800	−0.06915700
H	−0.78749300	−0.52584700	0.18439200
H	−0.06184000	0.94443300	0.18445800
H	0.84930600	−0.41881000	0.18440500

H_2O

O	0.00000000	0.00000000	0.11704100
H	0.00000000	0.76348700	−0.46816500
H	0.00000000	−0.76348700	−0.46816500

$EtOH_2^+$

C	−1.27517800	−0.27947300	−0.02593700
H	−1.32931100	−0.97383600	0.81631200
H	−1.31778700	−0.82180700	−0.97122900
H	−2.16100700	0.36205300	0.03247700
C	−0.08290700	0.62254800	0.05131800
H	0.03625300	1.28909300	−0.79959100
H	0.04437100	1.14226000	0.99918000
O	1.20049700	−0.27713400	−0.08665500
H	1.22188000	−1.03935100	0.52091300
H	2.05014000	0.20020600	−0.05711000

EtOH

C	1.22356400	−0.22355400	0.00000600
H	1.29174600	−0.85987300	−0.88568400
H	1.29173600	−0.85980600	0.88574000
H	2.07458400	0.46354700	−0.00001700
C	−0.08147300	0.54986700	−0.00000500
H	−0.13968500	1.19452500	0.88775100
H	−0.13965600	1.19446700	−0.88780300
O	−1.15506700	−0.39631100	−0.00000800
H	−1.99073300	0.07974300	0.00006500
